# Quantitative Susceptibility Mapping of Kidney Stones: An Ex Vivo MRI Phantom Study

**DOI:** 10.1002/mrm.70460

**Published:** 2026-06-05

**Authors:** Lion H. Mücke, Frederik B. Laun, Ruben de Figueiredo Cardoso, Guillaume Flé, Jennifer Lorenz, Alexander Fichte, Michael Uder, Nadine Bayerl, Jannis Hanspach

**Affiliations:** ^1^ Institute of Radiology Uniklinikum Erlangen, Friedrich‐Alexander‐Universität Erlangen‐Nürnberg Erlangen Germany; ^2^ Institute of Neuroradiology Uniklinikum Erlangen, Friedrich‐Alexander‐Universität Erlangen‐Nürnberg Erlangen Germany; ^3^ Department of Urology and Pediatric Urology Uniklinikum Erlangen, Friedrich‐Alexander‐Universität Erlangen‐Nürnberg Erlangen Germany; ^4^ Department of Nephrology and Hypertension Uniklinikum Erlangen, Friedrich‐Alexander‐Universität Erlangen‐Nürnberg Erlangen Germany

**Keywords:** kidney stones, phantom, quantitative susceptibility mapping, renal calcifications, renal calculi, renal MRI

## Abstract

**Purpose:**

To visualize and characterize the five most common kidney stone types based on their magnetic susceptibilities in MRI using QSM.

**Methods:**

Three water‐based agar phantoms were constructed, containing a total of 53 ex vivo kidney stones of varying types and sizes. Seven different MRI multi‐echo gradient echo sequences were employed at field strengths of 1.5 T and 3 T. Each acquisition was repeated three times. Susceptibility maps were reconstructed and mean susceptibility values calculated for the individual kidney stones (normalized to the agar gel). Five of the most common kidney stone types—carbonate apatite, calcium oxalate, uric acid, cystine, and struvite—were investigated with respect to different sequence parameters and field strengths.

**Results:**

All examined kidney stones were reliably visualized as diamagnetic using QSM. Overall, good repeatability was observed for the mean susceptibility values and standard deviations of the individual kidney stones. Mean susceptibility values were highly consistent across acquisitions at both field strengths for kidney stones belonging to the five major types with diameters larger than 3.0 mm (66% of all stones). The overall mean susceptibility values (± SD) were − 1.25 ± 0.16 ppm for carbonate apatite, −1.08 ± 0.13 ppm for calcium oxalate, −0.94 ± 0.17 ppm for uric acid, −1.32 ± 0.16 ppm for cystine, and −1.28 ± 0.28 ppm for struvite.

**Conclusion:**

Our data indicate that QSM is well‐suited to reliably visualize kidney stones and distinguish certain types among the five most common kidney stone compositions using MRI. This represents a crucial step toward in vivo imaging of suspected urolithiasis in clinical practice.

## 1 Introduction

Urolithiasis refers to the formation of solid stone‐like masses within the kidneys, commonly referred to as kidney stones, which may pass through the urinary tract [1, 2]. While non‐obstructing kidney stones are often asymptomatic, passage of a stone through the ureter can cause severe flank or pelvic pain—termed renal colic—depending on the site of obstruction [1, 2, 3]. It is estimated that approximately 1 in 11 people in the United States will experience a renal colic in their lifetime [2, 3]. Major risk factors for the formation of kidney stones are a family history of kidney stones, high body mass index, high blood pressure, metabolic disturbances, and kidney anatomy variants, such as horseshoe or pelvic kidneys [1, 2, 4, 5]. Possible complications of urolithiasis include infection, abscess formation, bleeding, and, in severe cases, urosepsis or acute kidney failure [1, 2].

Kidney stones typically form and grow as a result of urinary supersaturation, with the chemical composition of a stone reflecting the excess salts and metabolites of the urine [1, 2, 4]. Kidney stones can therefore be classified into several major types, with different chemical compositions and prevalence: calcium oxalate (60%–70% of all kidney stones) [1, 2, 6], calcium phosphate (1%–15%) [4, 7], uric acid (5%–10%) [1], cystine (1%–3%) [1, 2], struvite (1%–20%) [1, 2], and medication‐induced stones (approximately 1%) [1]. Struvite and cystine stones usually result from urinary tract infections and a genetic predisposition to cystinuria, respectively. In contrast, stones consisting of calcium oxalate, calcium phosphate, and uric acid are often caused by inadequate dietary habits or metabolic diseases [1, 2, 4, 5]. Identifying urine and stone chemistries is hence crucial for long‐term prevention and adequate diagnosis [2].

In clinical practice, non‐contrast abdominal CT is considered the reference standard for detecting kidney stones in vivo, due to its sensitivity and specificity of nearly 100% [1, 2, 3]. CT is highly effective for providing information on the size and location of most stone types, essential for treatment [1, 3]. However, CT may fail to visualize pure matrix stones consisting of proteinaceous material, as well as indinavir stones, which are common in HIV patients [1, 3]. Another disadvantage of abdominal CT is radiation exposure. Depending on the protocol, effective doses for abdominal CT in suspected renal colic range from 5 to 17 mSv [8, 9] for conventional‐dose CT and 1–3 mSv [10, 11] for low‐dose CT, posing a challenge for young patients and pregnant women [1, 3]. Given the high recurrence rate of kidney stones, at approximately 50% over 5 to 10 years, repeated CT imaging also increases lifetime cancer risk due to the accumulation of ionizing radiation, especially in young patients [3, 12, 13]. Ultrasound, in comparison, has lower sensitivity for determining stone location and size, but allows for easy and radiation‐free visualization of secondary effects related to urolithiasis, such as hydronephrosis, superimposed infection, or abscess formation [1, 3]. Therefore, ultrasound is widely used in patients with renal colic symptoms. As a further radiation‐free imaging modality, MRI provides superior abdominopelvic soft tissue information with respect to secondary effects, such as urinary tract dilation, inflammatory or ischemic changes, renal abscess, or adjacent edema [1, 13, 14]. Despite these advantages, conventional MRI has not yet proven feasible for urolithiasis imaging, as rapid signal decay means kidney stones are barely visible because of signal voids in T1‐, T2‐, and proton density‐weighted images [1, 13, 14, 15].

The MRI‐based contrast QSM has emerged as uniquely suited for the visualization of calcifications [16, 17, 18] and hemorrhages [16, 17, 18, 19], pathologies for which conventional MRI sequences often suffer from non‐specific presentation and low signal [1, 20, 21]. In QSM, the magnetic susceptibility—the degree to which a material responds to a magnetic field—is reconstructed from phase data and presented as susceptibility maps [21, 22, 23, 24]. While QSM has been predominantly applied in the brain [16, 17, 18, 21, 22, 23, 25], previous studies investigating abdominal and pelvic QSM have demonstrated its feasibility for detecting and displaying various conditions, including prostatic [26] and breast calcifications [27], carotid atherosclerotic plaques [28], skeletal bones [29, 30], and kidney fibrosis in patients with chronic kidney disease (CKD) [31]. Recently, Schumacher et al. [32] visualized two kidney stones of 9 and 10 mm using QSM in a study on the differentiation between hemorrhagic and dense proteinaceous cysts in patients with autosomal dominant polycystic kidney disease (ADPKD). However, because the chemical composition of the detected stones was unknown [32], it remains unclear whether all types can reliably be detected with QSM. Given that patients with urolithiasis appear to have an increased risk of CKD, especially those with cystine and struvite staghorn stones [2], further investigation of QSM presentations of different kidney stone types is necessary.

Therefore, the objective of this work was to visualize and characterize the five most common kidney stone types, with known chemical compositions and varying sizes, ex vivo using QSM.

## 2 Methods

### 2.1 Kidney Stone Population

A total of 53 kidney stones collected from 32 different patients were investigated. Of these, 12 patients provided a single kidney stone, while 19 provided two, and one patient provided three stones. Stone diameters d were calculated as $d=2\sqrt{A/\pi }$ from the axial stone cross‐section area A, with sizes ranging between 2.3 mm and 16.4 mm. The dimensions of the cross‐section area of each stone were measured manually with a ruler to the nearest 0.1 mm prior to phantom insertion. After completing the MRI measurements, all kidney stones were chemically analyzed using infrared spectroscopy (SYNLAB MVZ, Nuremberg, Germany) [4], destroying the samples in the process. Ultimately, 42 stones were classified as belonging to the five most common kidney stone types [1, 2, 4]: CaP (one pure carbonate apatite stone and two 80% carbonate apatite with 20% struvite), CaOx, (nine pure calcium‐oxalate‐monohydrate stones and four 60% calcium‐oxalate‐dihydrate with 40% calcium‐oxalate‐monohydrate), UA (all stones were 80% uric acid with 20% monoammoniumurate), CY (including three pure cystine stones and two 90% cystine with 10% calcium‐oxalate‐monohydrate), and ST (all stones were 70% struvite with 30% carbonate apatite). The remaining 11 stones were categorized as “mixed stones”, composed of varying mixtures of the major types, with no clear predominant allocation feasible. Details regarding the kidney stone population of the different types are denoted in Table 1, while the chemical compositions of all individual kidney stones are listed in the Table S1.

**TABLE 1:** Investigated kidney stone types with their respective number of individual stones, the range of diameters of the individual stones, and the number of kidney stones per type with diameters d>3mm used for quantitative evaluation.

Kidney stone type	Total number of stones	Diameter range in mm	Number of stones with d>3mm
CaP	3	3.9 to 5.0	2
CaOx	14	2.3 to 10.6	12
UA	12	2.3 to 10.1	9
CY	5	5.6 to 15.4	5
ST	8	2.3 to 16.4	7
Mixed	11	2.5 to 13.5	8

Abbreviations: CaOx, calcium oxalate; CaP, carbonate apatite; CY, cystine; Mixed, mixed stones; ST, struvite; UA, uric acid.

The kidney stone samples were fully anonymized residual materials from routine clinical care and would otherwise have been discarded. No patient identifiers or related clinical data were collected or analyzed. Formal institutional review board approval and informed consent were therefore not required as confirmed by the Ethics Committee of the Friedrich‐Alexander‐Universität Erlangen‐Nürnberg (reference number 26‐7‐ANF).

### 2.2 Phantom Preparation

**FIGURE 1:**
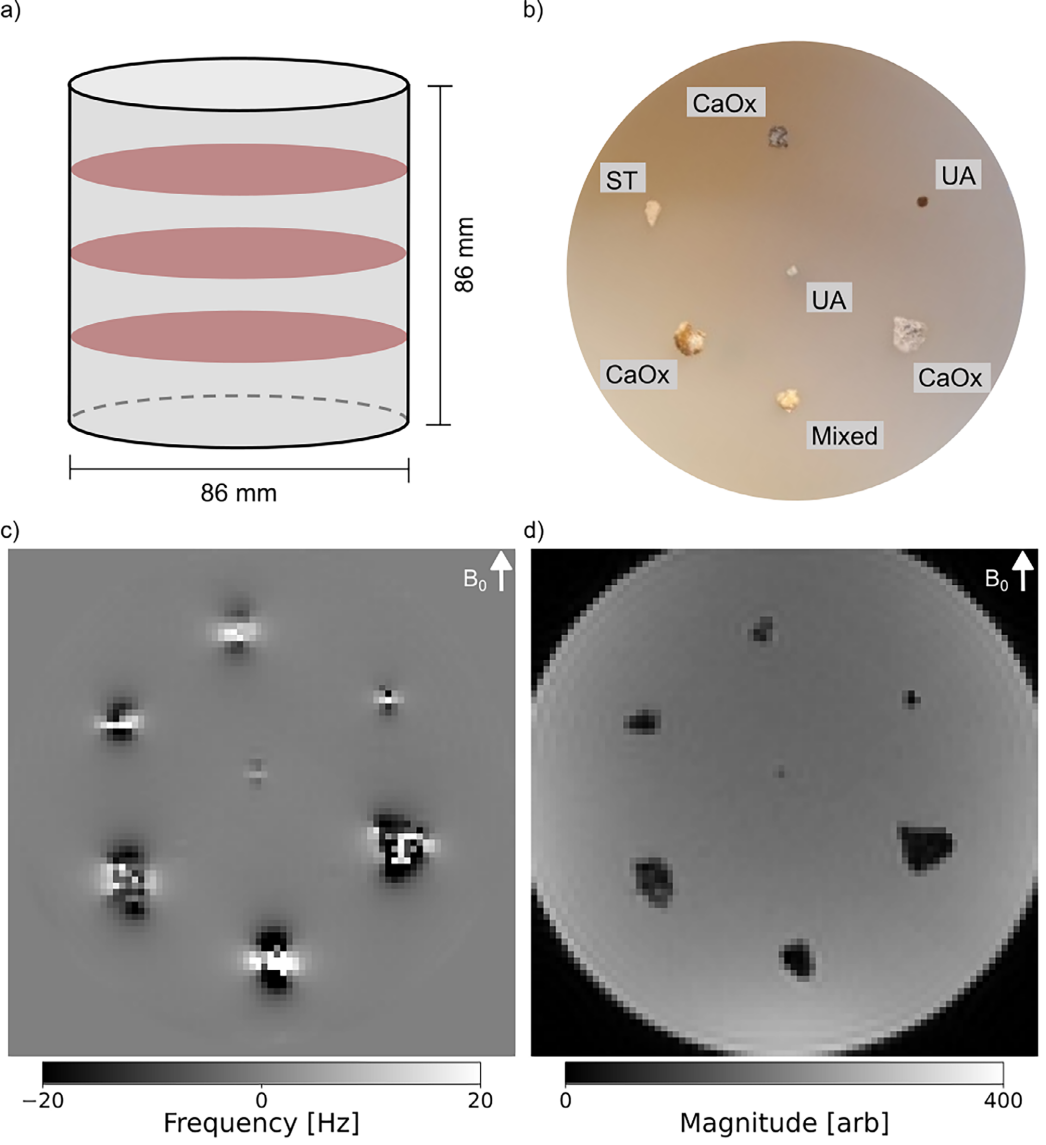
Kidney stone phantom. (a) Sketched phantom design with four layers of agar (gray) and three boundary layers, highlighting the locations of sealed‐in kidney stones (red) within a cylindrical phantom container (black). (b) Representative image of one embedded kidney stone layer of Phantom 2, consisting of 7 stones in a circular pattern with approximately equidistant spacing between the stones. The stone types are stated next to the stones. (c) Representative local field map of the kidney stone layer depicted in (b), acquired with the sequence 4TE‐Sola‐2. (d) Corresponding magnitude image of the first TE. The direction of the *B*_0_‐field is indicated in (c) and (d) by the white arrow. CaOx, calcium oxalate; Mixed, mixed stones; ST, struvite; UA, uric acid.

Three cylindrical phantoms were designed to fix the stones in place and consisted of 2 wt. % Agar‐Agar (Agar‐Agar Kobe 1 powder, Carl Roth GmbH + Co. KG, Karlsruhe, Germany) and 0.5 wt. % sodium chloride (NaCl, Carl Roth GmbH + Co. KG, Karlsruhe, Germany) [33, 34]. Three identical plastic cylindrical containers were used (polypropylene, height: 86 mm, diameter: 86 mm). The agar powder was slowly added and dissolved in a preheated saline solution at 90°C (NaCl mixed with tap water) under constant stirring. Each cylinder was filled by successively pouring four layers of agar solution, each 20 mm thick, after allowing the previous layer to solidify (Figure 1a). Kidney stones were placed directly on top of the solidified layer and were sealed by the next agar layer [34]. Care was taken to avoid air pockets or structural discontinuities within the phantom, simulating conditions similar to the natural occurrence of kidney stones in the body.

As depicted in Figure 1b, the kidney stones were arranged in a circular pattern with one additional stone in the center of each layer. The distance between adjacent stones was approximately 20 mm, with small variations resulting from the different stone sizes. The first phantom, “Phantom 1”, contained 11 kidney stones distributed over two enclosed boundary layers with five and six stones each. In each of “Phantom 2” and “Phantom 3”, 21 stones each were embedded, with seven stones per layer. The plastic cylinders were closed with a lid to minimize air contact, and the phantoms were stored in a refrigerator at approximately 4°C until the respective MR measurements.

### 2.3 Imaging Protocol

MRI scans were performed at two different field strengths, 1.5 T and 3 T (Magnetom Sola and Magnetom Vida, Siemens Healthineers, Erlangen, Germany), with 16‐channel ankle coils. Magnitude and phase data were acquired with a multi‐echo 3D gradient‐echo (GRE) sequence (volumetric interpolated breath‐hold examination sequence, VIBE) for each phantom separately. Data acquisition was performed with seven different parameter settings and with three measurement repetitions: 3TE‐Sola‐1, 4TE‐Sola‐2, 4TE‐Sola‐3, 5TE‐Sola‐4, 4TE‐Vida‐1, 4TE‐Vida‐2, 5TE‐Vida‐3. The seven acquisitions were based on the recommendations of the EMTP consensus for brain QSM [22]. Details on the differing sequence parameters TE, TR, voxel size, flip angle (FA), bandwidth (BW), number of averages, and acquisition time (TA) are listed in Table 2. All acquisitions used isotropic voxel sizes, an acquisition matrix of 112 × 128 × 112, monopolar echoes, and elliptical scanning with no further acceleration. Between repeated scans, the phantoms remained in the MR scanner. Given the known dependencies of reconstructed susceptibility maps on parameters such as first TE, last TE, voxel size, and SNR, we opted for a diverse set of acquisition settings to investigate and ensure the consistency of the QSM results [22, 23].

**TABLE 2:** Phantom data acquisition parameters of the seven different GRE sequence acquisition settings: TE, TR, voxel size, flip angle (FA), bandwidth (BW), number of averages (Avg), acquisition time (TA), and field strength.

Acquisition parameters	TE_1_/ΔTE/TE_max_ in ms	TR in ms	Voxel size in mm^3^	FA in °	BW in Hz/px	Avg	TA in min:s	Field strength
3TE‐Sola‐1	3.90/5.32/14.54	19	0.94 × 0.94 × 0.94	11	250	13	40:31	1.5 T
4TE‐Sola‐2	3.89/5.30/19.79	24	1.0 × 1.0 × 1.0	10	250	9	35:26	1.5 T
4TE‐Sola‐3	3.89/5.30/19.79	24	1.0 × 1.0 × 1.0	15	250	9	35:26	1.5 T
5TE‐Sola‐4	3.16/3.90/18.76	22	1.0 × 1.0 × 1.0	9	400	13	46:55	1.5 T
4TE‐Vida‐1	3.89/5.46/20.27	25	0.94 × 0.94 × 0.94	9	250	5	20:32	3 T
4TE‐Vida‐2	3.89/5.46/20.27	25	0.94 × 0.94 × 0.94	13	250	5	20:32	3 T
5TE‐Vida‐3	2.90/3.45/16.70	21	0.94 × 0.94 × 0.94	8	500	10	34:29	3 T

### 2.4 QSM Reconstruction

Reconstruction of the susceptibility maps was performed in MATLAB (R2022b; MathWorks, Natick, USA) utilizing the SEPIA toolbox [35] (version 1.2.1.1) [22]. First, masks of the phantoms were generated by thresholding the magnitude images and performing an erosion by three voxels. Phase unwrapping and echo phase combination were performed using ROMEO total field calculation [36] before removing background fields with the projection onto dipole fields (PDF) [19] method. Both algorithms were executed using the default SEPIA settings, except that phase offset correction was enabled in ROMEO and B1 field removal was disabled in the PDF step. The susceptibility maps were then reconstructed using Morphology‐Enabled Dipole inversion (MEDI + 0) [37] with a lambda factor of 50, which was determined by a broad parameter optimization. Here, the MEDI + 0 regularization enforcing homogeneous susceptibilities in the CSF was deactivated, rendering the dipole inversion effectively into the MEDI algorithm. Forward simulations of a numerical kidney stone phantom were performed to confirm the validity of this dipole inversion approach (see Supporting Information Simulations). All resulting susceptibility values were referenced to the mean susceptibility of the agar medium of the respective phantom.

### 2.5 Kidney Stone Evaluation

The volume of interest (VOI) of each kidney stone was manually segmented based on the first TE of the magnitude images using the Medical Imaging and Interaction Toolkit (MITK, DKFZ, Heidelberg, Germany) [38]. For each of the seven acquisition settings and three repetitions, mean susceptibility values and SDs were calculated for the VOIs of all 53 kidney stones. Based on the repeated measurements of mean susceptibility values per individual kidney stone, repeatability coefficients (RC) were estimated for each acquisition setting according to [39]: $\mathrm{RC}=1.96\cdot \sqrt{2s_{w}^{2}}$. Here, $s_{w}^{2}$ is the unbiased estimate of the within‐subject (means of square) variance, and the factor 1.96 results from the chosen confidence interval of 95% [39, 40]. Statistical analysis was performed in Python (version 3.11, scipy 1.16.2, pingouin 0.5.5). Further evaluation focused exclusively on stones belonging to the five major kidney stone types. Additionally, stones with diameters d≤3.0mm were excluded due to frequent partial volume effects and associated challenges in manual segmentation. The remaining data consisted of 35 kidney stones. For each kidney stone, mean susceptibility values were calculated from the VOIs obtained across all repetitions and respective acquisition settings. The Shapiro–Wilk test was used to assess the normality of data and thereby determine whether parametric and non‐parametric statistical analysis should be applied. Statistical testing of susceptibility differences between the major stone types was performed for both field strengths separately, excluding the stone type CaP due to its small sample size. For data acquired at 1.5 T, a Kruskal‐Wallis test was used, followed by a Dunn's post hoc test using the Holm‐Bonferroni method. For 3 T data, a Welch ANOVA test was applied, followed by a Games‐Howell post hoc test. To evaluate the effects of the acquisition settings and field strength on the measured susceptibility of the five major stone types, mean susceptibility values and SDs were calculated for the seven acquisitions based on the respective kidney stone population. Finally, for each of the five major kidney stone types, an overall mean susceptibility value was estimated.

## 3 Results

Across the seven different acquisition settings, all 53 investigated kidney stones were successfully visualized using QSM and presented as diamagnetic. Mean susceptibility values of the investigated stones ranged from χ=−0.43ppm to χ=−2.04ppm.

Independent of acquisition setting and kidney stone type, negligible signal was observed within kidney stone regions in the magnitude images, as shown in Figure 1d for Phantom 2. Stones with diameters d≤3.0mm frequently exhibited non‐zero, but still low signal. Furthermore, the local field maps obtained using the PDF method displayed high amounts of noise inside the VOI of most kidney stones with diameters d≥3.4mm (see Figure 1c).

**FIGURE 2:**
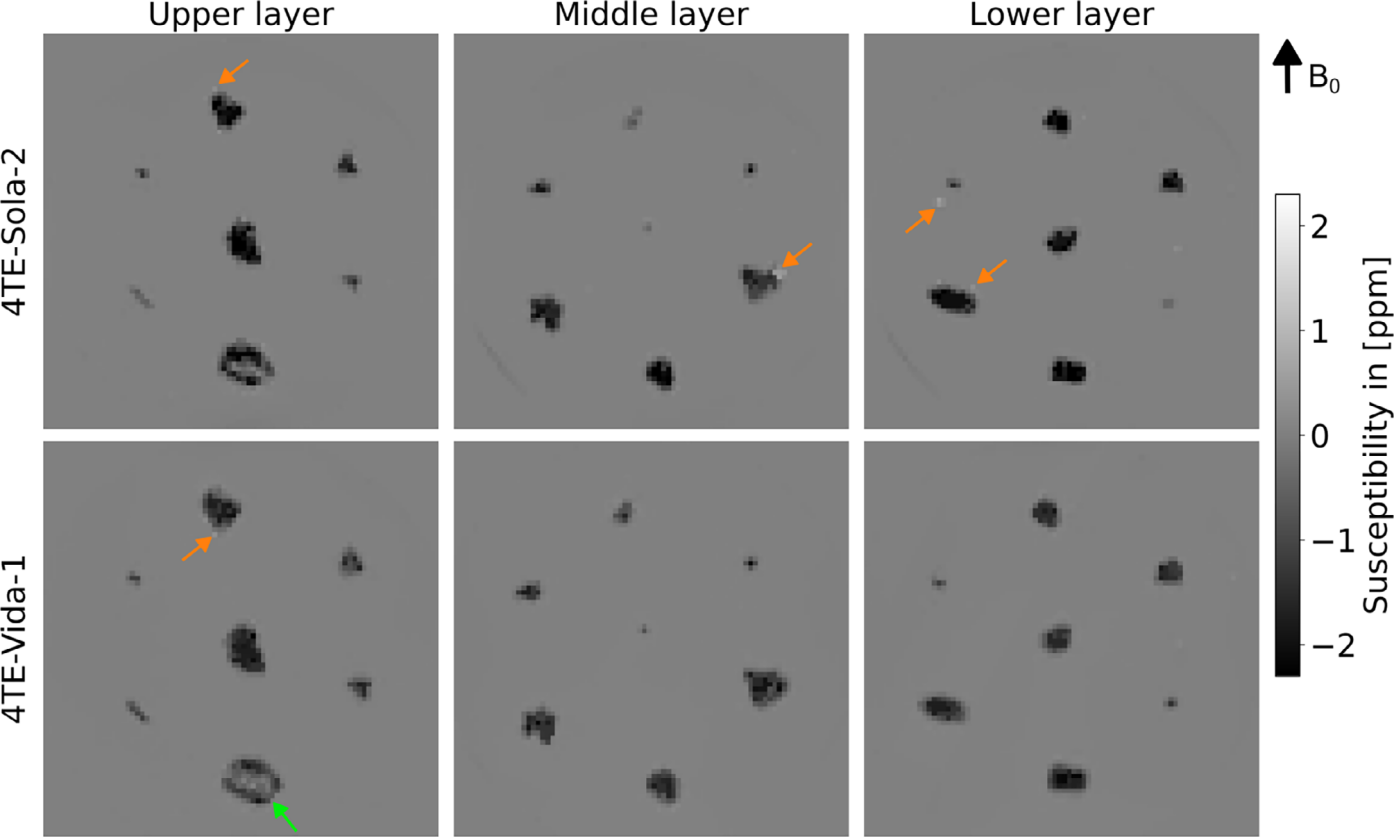
Representative susceptibility maps of Phantom 2 for the two acquisition settings, 4TE‐Sola‐2 at 1.5 T and 4TE‐Vida‐1 at 3 T, depicting three layers of kidney stones with seven stones each. All stone types presented as diamagnetic regions. Orange arrows point to examples of more pronounced paramagnetic regions at the boundary between the kidney stone and agar medium, which was present for most stones. The green arrow marks a stone with convexly arched shape, resulting in an interface between Agar‐Agar and kidney stone at the center of the stone in the depicted susceptibility map. The direction of the *B*_0_ field of both acquisitions is indicated by the black arrow.

Figure 2 depicts representative susceptibility maps of three stone layers embedded in Phantom 2 for the two acquisition settings 4TE‐Sola‐2 and 4TE‐Vibe‐1. The 21 different kidney stones in Phantom 2 can be clearly discriminated from the agar medium as diamagnetic regions at both 1.5 and 3 T. Representative susceptibility maps of Phantom 1 and Phantom 3 can be found in the Figure S1. Most kidney stones displayed noticeable inhomogeneities inside the VOI of the reconstructed susceptibility maps. The inhomogeneities appeared to be slightly stronger for data measured at 1.5 T field strength, but were independent of stone type or acquisition setting. For the majority of stones with diameters d≥3.4mm, there existed single voxels within the VOI of the susceptibility maps, which were roughly 1 ppm more diamagnetic than the remaining voxel values of the respective stone. The agar medium surrounding the kidney stones presented as homogeneous in the susceptibility maps, but single, isolated air bubbles were visible, despite careful phantom preparation, as small paramagnetic regions coinciding with small regions of no signal in the magnitude images. Based on visual inspection, the shapes, sizes, and positions of stones corresponded well between susceptibility maps and magnitude images at the first TE. Stones with diameters d≤3.0mm frequently appeared less diamagnetic. Multiple stones with diameters d≥3.4mm exhibited a weakly paramagnetic shell at the boundary between agar and stone, with more pronounced individual sub‐regions along a superior–inferior axis (orange arrows in Figure 2). However, these paramagnetic regions were not included in the VOI segmentations and thus did not affect quantitative evaluation. During visual assessment of the susceptibility maps, seven kidney stones seemed to display a composition of two compartments with slightly differing susceptibilities for the majority of acquisition settings. Of those, only two were classified as mixed stones, whereas four were of non‐pure composition within major types, and one stone was pure cystine.

**FIGURE 3:**
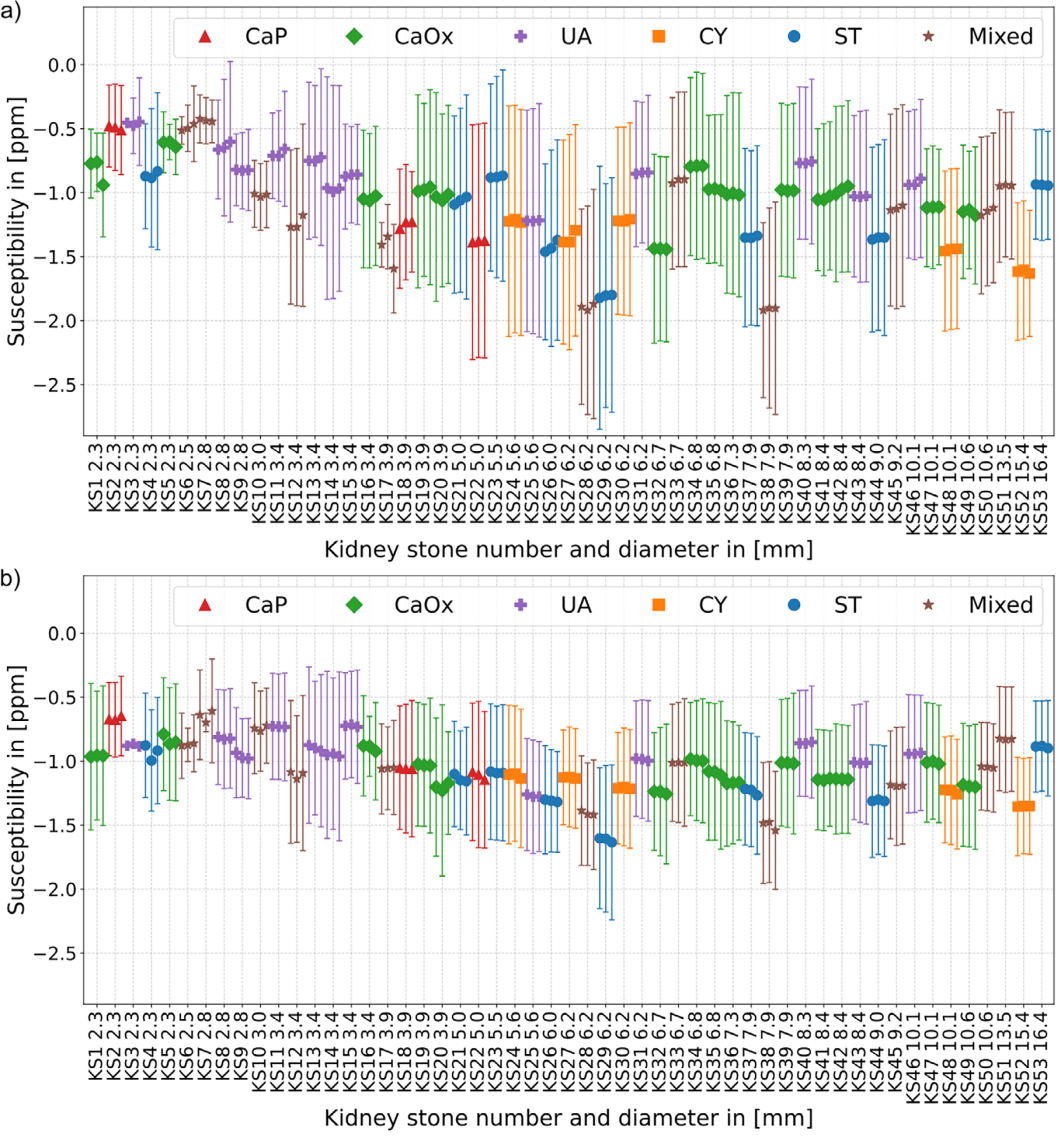
Mean susceptibility values and SD in ppm for the three measured repetitions of all 53 kidney stones, resulting from the representative sequences (a) 4TE‐Sola‐2 at 1.5 T and (b) 4TE‐Vida‐1 at 3 T. The stones are sorted by diameter in mm from smallest to largest along the x‐axis, where repetitions of individual kidney stones (KS) are clustered together and labeled with the same ID. Identically colored and shaped markers identify the five major kidney stone types, as well as mixed stones. CaOx, calcium oxalate; CaP, carbonate apatite; CY, cystine; Mixed, mixed stones; ST, struvite; UA, uric acid.

As depicted in Figure 3, the majority of kidney stones displayed similar mean susceptibility values and SD across three repetitions for the respective stone type and acquisition setting. For stones with diameters d≤3.4mm, isolated deviations in mean susceptibility and SD were observed (e.g., kidney stone KS17 in Figure 3a). Compared to larger stones of the same type, kidney stones with diameters d≤3.0mm often exhibited mean susceptibility values closer to 0 ppm. Overall, a reduction in SD was found for data acquired at 3 T field strength when compared to 1.5 T. Consistent with this observation, the RC of sequences acquired at 3 T ranged from 0.032 ppm to 0.056 ppm and thus showed better repeatability compared to sequences acquired at 1.5 T, which produced RCs between 0.084 ppm and 0.139 ppm (see Table 3). For the remaining five acquisition settings, the values of mean susceptibility and SD of all investigated stones can be found in the Figure S2. Group comparisons and summary statistics of kidney stone susceptibilities between variations of the acquisition parameter values first TE, ΔTE, TE_max_, FA, and field strength are presented in Figure S3 and Table S3.

**TABLE 3:** Repeatability coefficients (RC) for the susceptibility maps acquired by the seven investigated MRI acquisition settings.

Sequence	RC in ppm
3TE‐Sola‐1	0.139
4TE‐Sola‐2	0.084
4TE‐Sola‐3	0.086
5TE‐Sola‐4	0.105
4TE‐Vida‐1	0.032
4TE‐Vida‐2	0.052
5TE‐Vida‐3	0.056

**FIGURE 4:**
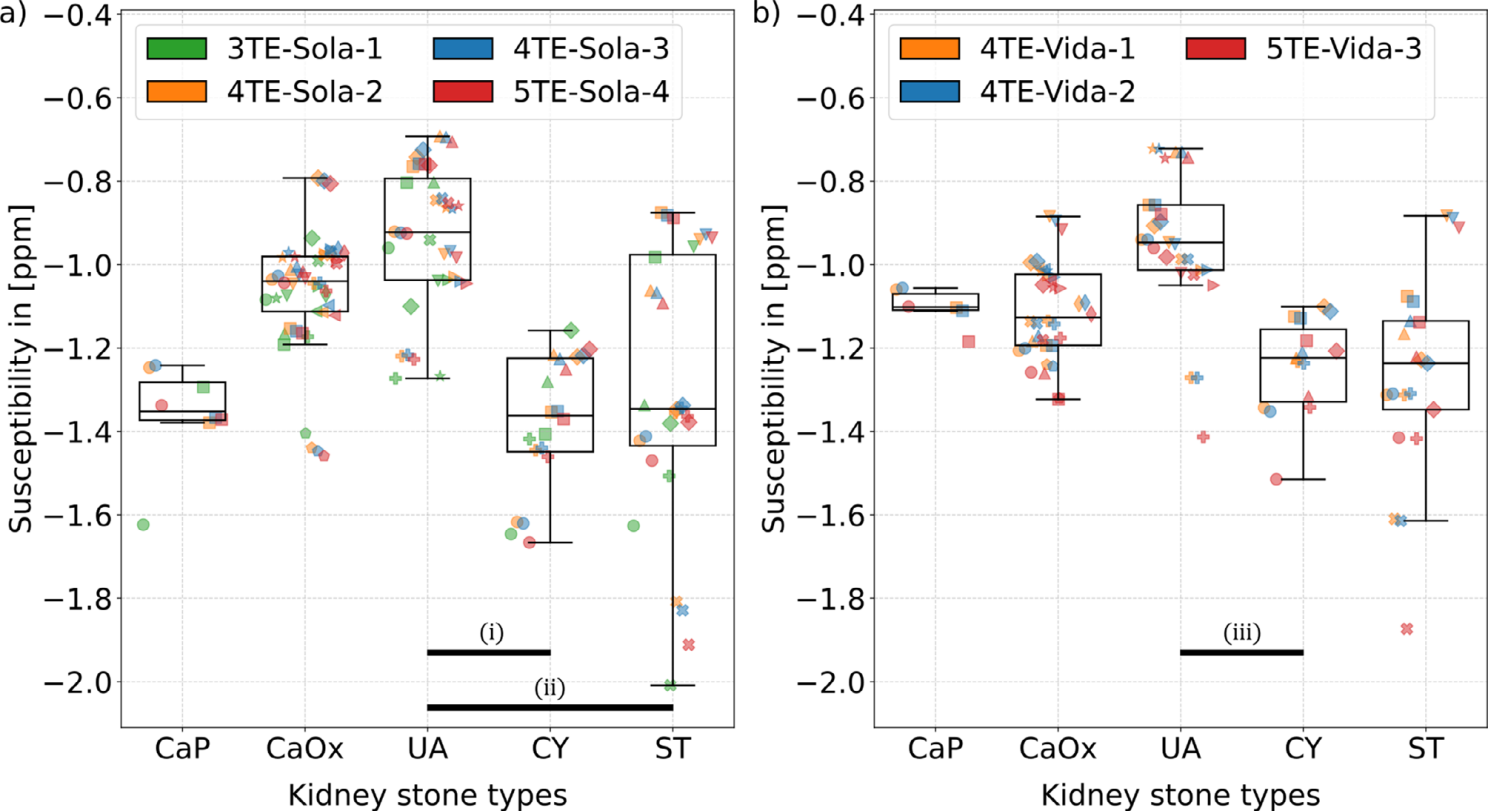
Group comparisons of magnetic susceptibility in ppm between the five major kidney stone types at (a) 1.5 T and (b) 3 T for stones with diameters d>3mm. The markers represent the mean susceptibility values of individual kidney stones across three repetitions for different sequences (see color legend). Identically shaped markers within one type represent the mean susceptibility values for different sequences of the same individual stone, while differently colored markers represent different measurement sequences. Statistically significant susceptibility differences are highlighted in black ($p_{\left(i\right)}=0.003$, $p_{\left(\mathrm{ii}\right)}=0.032$, $p_{\left(\mathrm{iii}\right)}=0.010$). Medians are provided per type, and whiskers depict 1.5 times the interquartile range (IQR). The IQRs are (a) $\mathrm{IQ}R_{\mathrm{CaP}}=0.09\;\mathrm{ppm}$, $\mathrm{IQ}R_{\text{CaOx}}=0.13\;\mathrm{ppm}$, $\mathrm{IQ}R_{\mathrm{UA}}=0.24\;\mathrm{ppm}$, $\mathrm{IQ}R_{\mathrm{CY}}=0.22\;\mathrm{ppm}$, $\mathrm{IQ}R_{\mathrm{ST}}=0.46\;\mathrm{ppm}$ and (b) $\mathrm{IQ}R_{\mathrm{CaP}}=0.04\;\mathrm{ppm}$, $\mathrm{IQ}R_{\text{CaOx}}=0.17\;\mathrm{ppm}$, $\mathrm{IQ}R_{\mathrm{UA}}=0.16\;\mathrm{ppm}$, $\mathrm{IQ}R_{\mathrm{CY}}=0.17\;\mathrm{ppm}$, $\mathrm{IQ}R_{\mathrm{ST}}=0.21\;\mathrm{ppm}$. CaOx, calcium oxalate; CaP, carbonate apatite; CY, cystine; ST, struvite; UA, uric acid.

Quantitative group comparisons between the susceptibilities of the five major kidney stone types are presented in Figure 4, together with mean susceptibility values of the 35 individual stones. Stones composed of UA appeared the least diamagnetic, showing greater distinction from CaOx and ST in data acquired at 3 T. Among CaP, CaOx, CY, and ST types, all stones showed a similar diamagnetic behavior. For all five major types, data acquired at 3 T resulted in a reduced spread of mean susceptibility values compared to 1.5 T field strength (see Figure 4). Individual kidney stones belonging to CaP, CaOx, UA, and CY types demonstrated largely good mean susceptibility reproducibility across different acquisition settings. In contrast, the ST type included multiple stones with a susceptibility difference slightly larger than 0.2 ppm between the most and least diamagnetic mean value. Statistical analysis of the individual kidney stones' susceptibility values yielded a significant difference for the stone types UA and CY at both 1.5 T (p=0.003) and 3 T (p=0.010), as well as between UA and ST types at 1.5 T (p=0.032). All calculated *p*‐values of the differences between the stone types can be found in Table S2.

**FIGURE 5:**
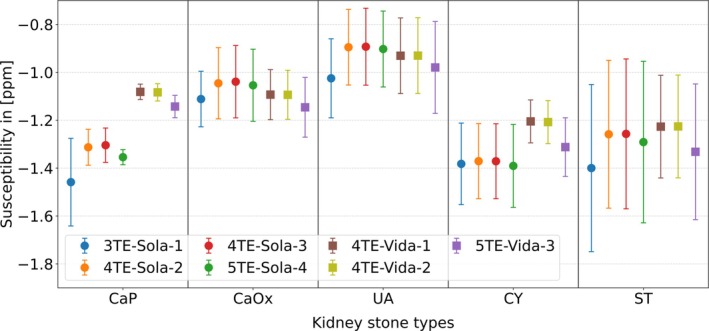
Comparison between the mean magnetic susceptibility of different sequences for the five major kidney stone types based on stones with diameters d>3mm: CaOx, calcium oxalate; CaP, carbonate apatite; CY, cystine; ST, struvite; UA, uric acid. Differently colored markers represent different sequences (see color legend); circular markers highlight sequences at 1.5 T, and squared markers represent sequences at 3 T. The displayed errors are SDs of the respective stone type and sequence.

Figure 5 compares the mean susceptibility values of the seven acquisition settings for stones of d≥3.4mm for each kidney stone type. UA exhibited the least diamagnetic mean susceptibilities among the five major types. Across field strengths, the acquisition settings 3TE‐Sola‐1 and 5TE‐Vida‐3—characterized by notably shorter last TEs—exhibited more diamagnetic mean values across the five major kidney stone types. Similarly, a slightly lower susceptibility value was observed for 5TE‐Sola‐4. Other than 3TE‐Sola‐1 and 5TE‐Vida‐3, the remaining five acquisition settings yielded similar mean susceptibilities for UA and ST stone types across both field strengths. For CaP and CY types, the mean values of data acquired at 3 T resulted in considerably less diamagnetic susceptibilities compared to 1.5 T data. In contrast, for CaOx stones, the susceptibilities of data acquired at 3 T were slightly more diamagnetic compared to the respective 1.5 T data. Acquisition settings differing only in FA yielded near‐identical susceptibilities across types. Except for CaP and UA stones, the SDs of the acquisition setting susceptibilities were generally larger at 1.5 T than at 3 T. Additionally, the large SD values observed for ST and CaP were consistent with the variability of individual stone susceptibilities depicted in Figure 4.

The overall mean susceptibility value and SD per major kidney stone type calculated across all acquisition settings and respective stones can be found in Table 4. Again, UA emerged as the least diamagnetic stone type, with $\chi_{\mathrm{UA}}=-0.94\pm 0.17\;\mathrm{ppm}$, while CY yielded the most diamagnetic overall susceptibility of $\chi_{\mathrm{CY}}=-1.32\pm 0.16\;\mathrm{ppm}$. The remaining kidney stone types resulted in $\chi_{\mathrm{CaP}}=-1.25\pm 0.16\;\mathrm{ppm}$ for CaP, $\chi_{\text{CaOx}}=-1.08\pm 0.13\;\mathrm{ppm}$ for CaOx, and $\chi_{\mathrm{ST}}=-1.28\pm 0.29\;\mathrm{ppm}$ for ST.

**TABLE 4:** Overall mean susceptibility values for the five major kidney stone types of stones with diameters d>3mm, together with the SDs of the respective kidney stone population. Additionally provided is the number of evaluated stones per type with diameters larger than d>3 mm.

Kidney stone type	Number of evaluated stones	Mean susceptibility in ppm
CaP	2	−1.25 ± 0.16
CaOx	12	−1.08 ± 0.13
UA	9	−0.94 ± 0.17
CY	5	−1.32 ± 0.16
ST	7	−1.28 ± 0.29

*Note:* Mean values were calculated across all sequences and repetitions of the respective stones.

Abbreviations: CaOx, Calcium oxalate; CaP, Carbonate apatite; CY, Cystine; ST, Struvite; UA, Uric acid.

## 4 Discussion

In this phantom study, QSM enabled direct and reliable visualization of kidney stones belonging to the five most common chemical compositions, using MRI at the two most widely used clinical field strengths, 1.5 T and 3 T [41]. Notably, high intra‐sequence repeatability and strong consistency between sequences were observed for the susceptibility values of individual kidney stones and across stone types when comparing seven different multi‐echo GRE sequence acquisition settings. As the phantom design simulated kidney stones submerged in a water‐based medium—similar to conditions in the body—the results of this study represent an important step toward the in vivo application of QSM for MRI‐based detection of kidney stones.

Our resulting susceptibility values of individual kidney stones (range: χ=−0.43ppm to χ=−2.04ppm) aligned well with results from previous studies: Schumacher et al. [32] reported on two in vivo kidney stones of unknown chemical composition with diameters of 9 mm and 10 mm, and found susceptibility values of approximately χ≈−1.5ppm using kidney parenchyma as reference tissue. Kan et al. [42] investigated five pseudo‐calcifications composed of hydroxyapatite (a subclass of calcium phosphate stones [7]) in a gelatin phantom, resulting in susceptibility values between approximately χ≈−0.5ppm and χ≈−2ppm with gelatin as reference tissue. Compared to the mean susceptibility values of the five major stone types denoted in Table 4, the slightly more diamagnetic susceptibility reported in vivo by Schumacher et al. [32] may be attributable to differences in reference tissues, phase data processing during QSM reconstruction, and sequence parameters. Our finding that kidney stones with diameters d≤3.0mm appeared less diamagnetic due to partial volume effects is consistent with observations for hydroxyapatite stones reported by Kan et al. [42] supporting our chosen size threshold for stone evaluation to avoid susceptibility bias derived from partial volume effects. While composition‐specific physical properties such as hardness and CT‐based radiodensity have been reported for different stone types, there are currently no reported composition‐specific magnetic susceptibility values for native kidney stones in the clinical imaging literature [24, 32]. Ringden et al. [43] reported uric acid as the least hard and cystine as among the hardest stone types, with carbonate apatite, calcium oxalate, and struvite exhibiting intermediate values. Accordingly, uric acid stones were also reported to have the lowest radiodensity. However, cystine exhibited radiodensity values comparable to struvite and carbonate apatite, while calcium oxalate stones demonstrated the highest radiodensity [44, 45]. Our findings therefore suggest that kidney stones' magnetic susceptibility may not scale directly with radiodensity alone, but may additionally be influenced by differences in composition and microstructure.

Although UA emerged as the least diamagnetic kidney stone type and showed significant susceptibility differences from cystine and struvite, the small sample sizes of the five major stone types (n<20) undermined the validity of the statistical analysis [46]. Increasing the number of data points, for example by using individual acquisition setting measurements instead of mean susceptibility values per stone, would also violate the precondition of uncorrelated data [47]. Future studies including a larger number of kidney stones per type could investigate robust statistical differentiation between stone types using QSM.

The susceptibility values reported in this study present reasonable estimates, albeit the exact voxel values of the investigated kidney stones should be interpreted with caution. QSM reconstruction artifacts are frequently encountered in bone regions [29, 48], air inclusions [48, 49, 50], and intracerebral hemorrhages [51, 52, 53, 54], which are characterized by low signal and large susceptibilities in MRI, similar to kidney stones [24, 32]. Forward simulations performed in Supporting Information Simulations showed that the utilized dipole inversion yielded accurate and reliable mean susceptibility values for kidney stones containing severe simulated gaussian noise within stone regions in the local field map, when compared to the ground truth of the simulation. However, in these forward simulations, strong susceptibility inhomogeneities within stone regions resulted in large standard deviations for kidney stone susceptibilities. Similarly, the noticeable inhomogeneities observed within the VOI of most kidney stones in the susceptibility maps of this phantom study did presumably not reflect the underlying susceptibility distribution inside the respective stones, but rather resulted from propagated phase noise. This particularly affected the identification and visual interpretation of mixed kidney stones in this study, since the reconstructed susceptibility maps may not accurately represent the true, potentially heterogeneous, internal chemical structure [2, 4]. Therefore, the apparent presence of two compartments with slightly differing susceptibilities, which were observed in six of the 40 non‐pure kidney stones, should be considered with caution. While QSM approaches like piece‐wise constant regularized dipole inversion [55] could reduce propagated phase noise by enforcing uniform susceptibility values within each kidney stone, their applicability is limited for in vivo kidney QSM. Future work may instead utilize a total field inversion [52, 53] based QSM reconstruction algorithm, which has shown good applicability in bone [30] and hemorrhage [28, 52, 53] regions.

For background field removal, PDF was chosen, as it has been proven to be reliable in regions with large susceptibilities and low signal [29, 56]. Furthermore, Dimov et al. [29] demonstrated the feasibility of in vivo bone QSM by combining UTE with a multi‐TE GRE sequence. Since calcium hydroxyapatite is a major chemical component of bone [57], applying UTE to kidney stones could improve the reconstructed susceptibility maps through the acquisition of non‐vanishing phase information in stone regions with similarly ultrashort T2* relaxation times ($T2_{\text{bone}}^{*}\approx 0.3\;\mathrm{ms}$) [58]. Indeed, both Ibrahim et al. [15] and Yassin et al. [59] successfully acquired conventional MR images of ex vivo kidney stones using UTE radial sequences. For clinical kidney QSM, however, the short last TE and the low number of TEs in UTE sequences are likely to limit accuracy of kidney soft tissue susceptibilities [22, 31]. In contrast, the TEs and QSM reconstruction method used in our study appear feasible for simultaneous in vivo visualization of kidney stones and soft tissues in susceptibility maps. Additionally, Cartesian multi‐TE GRE sequences are widely available on scanners from all major manufacturers and may therefore facilitate faster integration into clinical routine [18, 22, 32].

Across the seven investigated multi‐TE GRE acquisition settings, the overall effect sizes on kidney stone susceptibilities for the different field strengths of 1.5 T and 3 T, as well as variations of the sequence parameters first TE, ΔTE, and FA, which are deemed crucial for QSM [22], remained small (see Figure S3 and Table S3). The largest effect was observed for variations of the last TE, where decreasing values of the last TE shifted the susceptibilities of most kidney stones toward more diamagnetic values, but did not substantially alter the relative susceptibilities between different stone types. This overall consistency is promising for optimizing the acquisition parameters for in vivo QSM of the kidney, where TEs must be tailored to account for chemical fat shifts, and the last TE should match the T2* relaxation time of kidney soft tissue [22, 24, 60]. Previous in vivo kidney QSM studies—examining oxygen consumption in volunteers consuming caffeine [61], kidney fibrosis in CKD patients [31], and the differentiation of hemorrhagic from dense proteinaceous cysts in ADPKD patients [32]—have employed breath‐hold techniques and fast multi‐TE GRE sequences with a last TE between 6.7 ms and 15.0 ms to compensate for breathing motion artifacts. Further phantom and in vivo studies should investigate the exact effect of these shorter last TEs on the susceptibility values of kidney stones, as well as underestimations expected for considerably larger voxel sizes (e.g., 1.64 × 1.64 × 3.0 mm^3^ in Bechler et al.) [31, 62, 63].

The design of our three phantoms allowed for direct contact between the kidney stones and agar medium without visible air bubbles, thereby mimicking their natural occurrence in water‐based tissues and avoiding the use of obstructing instruments such as plastic vials. Similar interface‐free phantom designs have been employed in several QSM phantom studies [34, 64, 65, 66]. Sealing the kidney stones during phantom creation enabled the liquified agar solution to surround and fit almost perfectly to their shape, thus minimizing the risk of air bubble formation. Although no air bubbles were visible during phantom creation, small signal void regions in magnitude images with high paramagnetic susceptibilities suggested sporadic entrapped air bubbles, all located far from kidney stone regions. Furthermore, some kidney stones showed rehydration effects during phantom creation, where stones started to emit air bubbles when fully surrounded by the agar solution. Dawson et al. [14] submerged their 141 investigated kidney stones in water for 48 h before MRI measurements; however, neither Kan et al. [42] nor Ibrahim et al. [15] reported preceding rehydration or similar observations in their MRI kidney stone phantom studies. While paramagnetic regions, located at the boundary between kidney stones and agar medium, may have resulted from tiny amounts of air trapped at the stone edge, it is more likely these effects were artifacts of the QSM reconstruction. A particular indication for these artifacts being related to the QSM reconstruction is the preference direction (superior–inferior in Figure 2) observed for strongly pronounced paramagnetic regions at the stone‐agar boundary. Although variations in the magnitude signal between different stones complicated the optimization of the VOI segmentations, the paramagnetic artifacts seem to be merely cosmetic, as their paramagnetic values were located outside of the evaluated stone volumes. Nonetheless, using a QSM reconstruction algorithm capable of handling a large range of susceptibility values, such as total field inversion [52, 53], may improve the visual presentation of these paramagnetic artifacts.

QSM may represent an MRI‐based future alternative to CT for the detection of kidney stones [24, 32]. This would be useful, not only for the simultaneous acquisition of high‐quality abdominal images to detect secondary pathologies or surgical emergencies presenting with symptoms similar to urolithiasis [1, 13], but also for radiation‐free monitoring of kidney stone expulsion and detection in pregnant, younger, and recurrent patients. Urolithiasis is also one of the most common clinically relevant complications associated with ADPKD [67], where QSM can also identify cystic hemorrhages [32], which are markers for rapid CKD progression [68, 69]. The large voxel sizes commonly used for breath‐hold sequences in kidney QSM may limit the detection of small kidney stones in vivo [31, 32, 61]. However, monitoring and the acquisition of soft tissue images are particularly important for larger stones with diameters above d>5mm, which often require invasive intervention, such as drainage via ureteric stent, fragmentation using extracorporeal shock wave lithotripsy or endoscopic laser lithotripsy, interventional percutaneous nephrostomy, or surgery [2, 4, 70]. Furthermore, the differentiation between venous calcification (i.e., phlebolith) and a kidney stone can be challenging on CT [70]. The acquisition of high‐quality soft tissue images in combination with kidney stone detection using QSM might prove superior in this regard, but in vivo studies are required to confirm this.

One main limitation of this work is that all 53 investigated kidney stones were destroyed during infrared spectroscopy chemical analysis, preventing any further measurements on the same samples. Infrared spectroscopy is widely regarded as the reference standard for kidney stone composition analysis and recommended according to the European Association of Urology guidelines [71], due to its good sensitivity and reproducibility, high availability, speed, minimal sample requirements and ability to reliably identify individual crystalline components as well as mixed and rare stone types [4, 14, 71, 72, 73]. Future work should investigate alternative, non‐destructive reference methods such as X‐ray diffraction, which requires larger sample volumes, longer analysis times, and specialized staff [72]. Dawson et al. [14] used shaved‐off samples instead of entire stones for infrared spectroscopy. However, this approach might yield uncertain chemical analysis estimations, given the potentially inhomogeneous mixtures of multiple chemical components within a single stone. A further limitation is that the kidney stones investigated in this study were a convenience sample, based on availability. For this reason, no CT‐negative kidney stones, such as indinavir stones (≈ 53% of all medication‐induced stones) from HIV patients [1], were included. Also, few stones were included of cystine, struvite, and especially calcium phosphate types. Additionally, the sample included no brushite stone, which accounts for about 25% of all calcium phosphate stones as the third main subclass, besides carbonate apatite and hydroxyapatite [7, 74]. Brushite stones are of particular interest with regard to treatment planning, since they are resistant to extracorporeal shock wave lithotripsy and often require repeated interventions [2]. For future studies, comparing the magnetic susceptibility of these infrequent kidney stone types using QSM, and validating the resulting susceptibilities with alternative methods such as a superconducting quantum interference device [75], would be of great interest.

Finally, our results are limited to phantom measurements, as no in vivo data from kidney stone patients were acquired. Nevertheless, our ex vivo results demonstrate the feasibility of QSM to visualize reliably at least 95% [1, 4, 7, 74] of all kidney stone types, establishing the foundation for a systematic patient study to evaluate the feasibility and limitations of QSM kidney stone detection in vivo. As previous kidney QSM studies have employed breath‐hold multi‐TE GRE sequences with substantially larger voxel sizes [31, 32, 61], a systematic investigation on the minimum detectable size of kidney stones using QSM at in vivo resolutions would be the next logical step toward translating this technique into clinical practice. Ultimately, future studies should investigate whether QSM can reliably detect kidney stones in vivo, which would represent an important step toward establishing this approach as a radiation‐free alternative for the diagnosis and follow‐up of urolithiasis patients.

## 5 Conclusions

This phantom study demonstrated that QSM can reliably visualize kidney stones composed of calcium oxalate, carbonate apatite, uric acid, cystine, and struvite as diamagnetic regions in water‐based surroundings using MRI at both 1.5 and 3 T field strength. Furthermore, susceptibility values of these five major stone types showed good consistency across varying sequence parameters of the widely available multi‐TE 3D GRE sequence, as well as high test–retest repeatability. Using QSM to directly and reliably visualize a presumed 95% of all kidney stones [1, 4, 7, 74], as well as potentially differentiate uric acid from cystine and struvite types, marks a crucial step toward an MRI‐based and radiation‐free alternative to unenhanced CT for in vivo kidney stone detection and imaging of urolithiasis patients in clinical practice.

## Funding

This work was supported by Interdisciplinary Center for Clinical Research (IZKF), Uniklinikum Erlangen, Laboratory Rotation, P125; Deutsche Forschungsgemeinschaft, Project Number 500888779/RU5534.

## Supporting information


**Figure S1:** Representative susceptibility maps (first repetition) of (a) the four acquisition settings at 1.5 T for Phantom 1 (11 kidney stones distributed over two layers); (b) the three acquisition settings at 3 T for Phantom 1; (c) the four acquisition settings at 1.5 T for Phantom 2 (21 kidney stones distributed over three layers); (d) the three acquisition settings at 3 T for Phantom 2; (e) the four acquisition settings at 1.5 T for Phantom 3 (21 kidney stones distributed over three layers); (f) the three acquisition settings at 3 T for Phantom 3. The direction of the B_0_‐field of the respective acquisitions is indicated by a black arrow.
**Figure S2:** Mean susceptibility values and SD in ppm for the three measured repetitions of all 53 kidney stones, resulting from the acquisition settings (a) 3TE‐Sola‐1; (b) 4TE‐Sola‐3; (c) 5TE‐Sola‐4; (d) 4TE‐Vida‐2; and (e) 5TE‐Vida‐3. The stones are sorted by diameter in (mm) from smallest to largest along the x‐axis, where repetitions of individual kidney stones (KS) are clustered together and labeled with the same ID. Identically colored and shaped markers identify the five major kidney stone types, as well as mixed stones. CaOx, calcium oxalate; CaP, carbonate apatite; CY, cystine; Mixed, mixed stones; ST, struvite; UA, uric acid.
**Figure S3:** Mean susceptibility values in ppm of the 53 individual kidney stones in blue for the different acquisition parameter values of the seven acquisition settings on the x‐axis for the acquisition parameters (a) first TE (TE1), (b) TE spacing (ΔTE), (c) last TE (TEmax), (d) flip angle (FA), (e) field strength. A linear least squares fit applied to the susceptibilities of each acquisition parameter is depicted in orange, with the slope, intercept and coefficient of determination (*R* ^2^) denoted at the top of each parameter analysis subfigure. Statistically significant differences between susceptibilities of the respective acquisition parameter values are highlighted in black (*p<0.01). Effects of different acquisition parameter values on the kidney stone susceptibilities of the 53 kidney stones were quantified by applying a Friedman test for each acquisition parameter with more than two different parameter values, followed by a Wilcoxon signed‐rank test using the Holm‐Bonferroni method. For exactly two different acquisition parameter values, a Wilcoxon signed‐rank test was calculated. All *p*‐values are listed in Table S3.
**Table S1:** Investigated kidney stones (KS) with their respective diameter in mm, chemical composition determined by infrared spectroscopy and allocated type in this study. CaOx, calcium oxalate; CaP, carbonate apatite; CY, cystine; Mixed, mixed stones; ST, struvite; UA, uric acid.
**Table S2:** Resulting *p*‐value matrices from statistical post hoc analysis for susceptibility differences between the major kidney stone types CaOx, UA, CY, and ST resulting from statistical analysis of data at (a) 1.5 T, (b) 3 T, and (c) both field strengths together. The data of each stone type consisted of the mean susceptibility values per individual kidney stone calculated from the three repetitions and respective acquisition settings. Significant differences (p<0.05) are highlighted in red. CaP was excluded due to its small sample size. CaOx, calcium oxalate; CaP, carbonate apatite; CY, cystine; Mixed, mixed stones; ST, struvite; UA, uric acid.
**Table S3:** Resulting *p*‐value matrices from statistical post hoc analysis of differences between susceptibility values of the 53 individual kidney stones at the different acquisition parameter values utilized in the seven acquisition settings, together with the median susceptibility values (Median χ) for each acquisition parameter setting. The *p*‐value matrices are given for the parameters (a) first TE (TE_1_), (b) TE spacing (ΔTE), (c) last TE (TE_max_), (d) flip angle (FA), (e) field strength. Significant differences (p<0.05) are highlighted in red.

## Data Availability

The data that support the findings of this study are available from the corresponding author upon reasonable request.
