# Supplementary material for: Quantitative Susceptibility Mapping of Kidney Stones: An Ex Vivo MRI Phantom Study
Source: Magn Reson Med. 2026 Jun 5;96(4):1846–59. doi: 10.1002/mrm.70460 (PMC13419359; doi:10.1002/mrm.70460)
Supplement: Supplementary file 1 — Figure S1: Representative susceptibility maps (first repetition) of (a) the four acquisition settings at 1.5 T for Phantom 1 (11 kidney stones distributed over two layers); (b) the three acquisition settings at 3 T for Phantom 1; (c) the four acquisition settings at 1.5 T for Phantom 2 (21 kidney stones distributed over three layers); (d) the three acquisition settings at 3 T for Phantom 2; (e) the four acquisition settings at 1.5 T for Phantom 3 (21 kidney stones distributed over three layers); (f) the three acquisition settings at 3 T for Phantom 3. The direction of the B0‐field of the respective acquisitions is indicated by a black arrow. Figure S2: Mean susceptibility values and SD in ppm for the three measured repetitions of all 53 kidney stones, resulting from the acquisition settings (a) 3TE‐Sola‐1; (b) 4TE‐Sola‐3; (c) 5TE‐Sola‐4; (d) 4TE‐Vida‐2; and (e) 5TE‐Vida‐3. The stones are sorted by diameter in (mm) from smallest to largest along the x‐axis, where repetitions of individual kidney stones (KS) are clustered together and labeled with the same ID. Identically colored and shaped markers identify the five major kidney stone types, as well as mixed stones. CaOx, calcium oxalate; CaP, carbonate apatite; CY, cystine; Mixed, mixed stones; ST, struvite; UA, uric acid. Figure S3: Mean susceptibility values in ppm of the 53 individual kidney stones in blue for the different acquisition parameter values of the seven acquisition settings on the x‐axis for the acquisition parameters (a) first TE (TE1), (b) TE spacing (ΔTE), (c) last TE (TEmax), (d) flip angle (FA), (e) field strength. A linear least squares fit applied to the susceptibilities of each acquisition parameter is depicted in orange, with the slope, intercept and coefficient of determination (R 2) denoted at the top of each parameter analysis subfigure. Statistically significant differences between susceptibilities of the respective acquisition parameter values are highlighted in black (*p<0.01). Effects o [file MRM-96-1846-s001.pdf]

SUPPORTING INFORMATION TABLE S1: Investigated kidney stones (KS) with their respective diameter in [mm], chemical composition determined by infrared spectroscopy and allocated type in this study. CaP = carbonate apatite, CaOx = calcium oxalate, UA = uric acid, CY = cystine, ST = struvite, Mixed = mixed stones.

Kidney stone ID	Diameter [mm]	Chemical composition	Allocated type
KS1	2.3	100% Calcium-Oxalate-Monohydrate (Whewellite)	CaOx
KS2	2.3	100% Carbonate Apatite	CaP
KS3	2.3	80% Uric Acid, 20% Monoammoniumurate	UA
KS4	2.3	70% Struvite, 30% Carbonate Apatite	ST
KS5	2.3	100% Calcium-Oxalate-Monohydrate (Whewellite)	CaOx
KS6	2.5	70% Calcium-Oxalate-Monohydrate (Whewellite), 30% Uric Acid	Mixed
KS7	2.8	55% Carbonate Apatite, 30% Calcium-Oxalate-Dihydrate (Weddellite), 15% Calcium-Oxalate-Monohydrate (Whewellite)	Mixed
KS8	2.8	80% Uric Acid, 20% Monoammoniumurate	UA
KS9	2.8	80% Uric Acid, 20% Monoammoniumurate	UA
KS10	3.0	50% Uric Acid, 50% Carbonate Apatite	Mixed
KS11	3.4	80% Uric Acid, 20% Monoammoniumurate	UA
KS12	3.4	40% Calcium-Oxalate-Monohydrate (Whewellite), 40% Struvite, 20% Carbonate Apatite	Mixed
KS13	3.4	80% Uric Acid, 20% Monoammoniumurate	UA
KS14	3.4	80% Uric Acid, 20% Monoammoniumurate	UA
KS15	3.4	80% Uric Acid, 20% Monoammoniumurate	UA
KS16	3.4	100% Calcium-Oxalate-Monohydrate (Whewellite)	CaOx
KS17	3.9	60% Calcium-Oxalate-Dihydrate (Weddellite), 25% Carbonate Apatite, 15% Calcium-Oxalate-Monohydrate (Whewellite)	Mixed
KS18	3.9	80% Carbonate Apatite, 20% Struvite	CaP
KS19	3.9	100% Calcium-Oxalate-Monohydrate (Whewellite)	CaOx
KS20	3.9	100% Calcium-Oxalate-Monohydrate (Whewellite)	CaOx
KS21	5.0	70% Struvite, 30% Carbonate Apatite	ST
KS22	5.0	80% Carbonate Apatite, 20% Struvite	CaP
KS23	5.5	70% Struvite, 30% Carbonate Apatite	ST
KS24	5.6	100% Cystine	CY
KS25	5.6	80% Uric Acid, 20% Monoammoniumurate	UA
KS26	6.0	70% Struvite, 30% Carbonate Apatite	ST
KS27	6.2	10% Calcium-Oxalate-Monohydrate (Whewellite), 90% Cystine	CY

KS28	6.2	55% Carbonate Apatite, 30% Calcium-Oxalate-Dihydrate (Weddellite) 30%, 15% Calcium-Oxalate-Monohydrate (Whewellite)	Mixed
KS29	6.2	70% Struvite, 30% Carbonate Apatite	ST
KS30	6.2	100% Cystine	CY
KS31	6.2	80% Uric Acid, 20% Monoammoniumurate	UA
KS32	6.7	60% Calcium-Oxalate-Dihydrate (Weddellite), 40% Calcium-Oxalate-Monohydrate (Whewellite)	CaOx
KS33	6.7	70% Calcium-Oxalate-Dihydrate (Weddellite), 30% Carbonate Apatite	Mixed
KS34	6.8	100% Calcium-Oxalate-Monohydrate (Whewellite)	CaOx
KS35	6.8	100% Calcium-Oxalate-Monohydrate (Whewellite)	CaOx
KS36	7.3	100% Calcium-Oxalate-Monohydrate (Whewellite)	CaOx
KS37	7.9	70% Struvite, 30% Carbonate Apatite	ST
KS38	7.9	40% Calcium-Oxalate-Monohydrate (Whewellite), 40% Struvite, 20% Carbonate Apatite	Mixed
KS39	7.9	100% Calcium-Oxalate-Monohydrate (Whewellite)	CaOx
KS40	8.3	80% Uric Acid, 20% Monoammoniumurate	UA
KS41	8.4	100% Calcium-Oxalate-Monohydrate (Whewellite)	CaOx
KS42	8.4	60% Calcium-Oxalate-Dihydrate (Weddellite), 40% Calcium-Oxalate-Monohydrate (Whewellite)	CaOx
KS43	8.4	80% Uric Acid, 20% Monoammoniumurate	UA
KS44	9.0	70% Struvite, 30% Carbonate Apatite	ST
KS45	9.2	60% Calcium-Oxalate-Monohydrate (Whewellite), 30% Struvite, 10% Carbonate Apatite	Mixed
KS46	10.1	80% Uric Acid, 20% Monoammoniumurate	UA
KS47	10.1	60% Calcium-Oxalate-Dihydrate (Weddellite), 40% Calcium-Oxalate-Monohydrate (Whewellite)	CaOx
KS48	10.1	90% Cystine, 10% Calcium-Oxalate-Monohydrate (Whewellite)	CY
KS49	10.6	60% Calcium-Oxalate-Dihydrate (Weddellite), 40% Calcium-Oxalate-Monohydrate (Whewellite)	CaOx
KS50	10.6	70% Calcium-Oxalate-Monohydrate (Whewellite), 30% Uric Acid	Mixed
KS51	13.5	70% Uric Acid, 30% Cystine	Mixed
KS52	15.4	100% Cystine	CY
KS53	16.4	70% Struvite, 30% Carbonate Apatite	ST

SUPPORTING INFORMATION FIGURE S1: Representative susceptibility maps (first repetition) of **a)** the four acquisition settings at 1.5 T for Phantom 1 (11 kidney stones distributed over two layers); **b)** the three acquisition settings at 3 T for Phantom 1; **c)** the four acquisition settings at 1.5 T for Phantom 2 (21 kidney stones distributed over three layers); **d)** the three acquisition settings at 3 T for Phantom 2; **e)** the four acquisition settings at 1.5 T for Phantom 3 (21 kidney stones distributed over three layers); **f)** the three acquisition settings at 3 T for Phantom 3. The direction of the B_0-field of the respective acquisitions is indicated by a black arrow.

a)

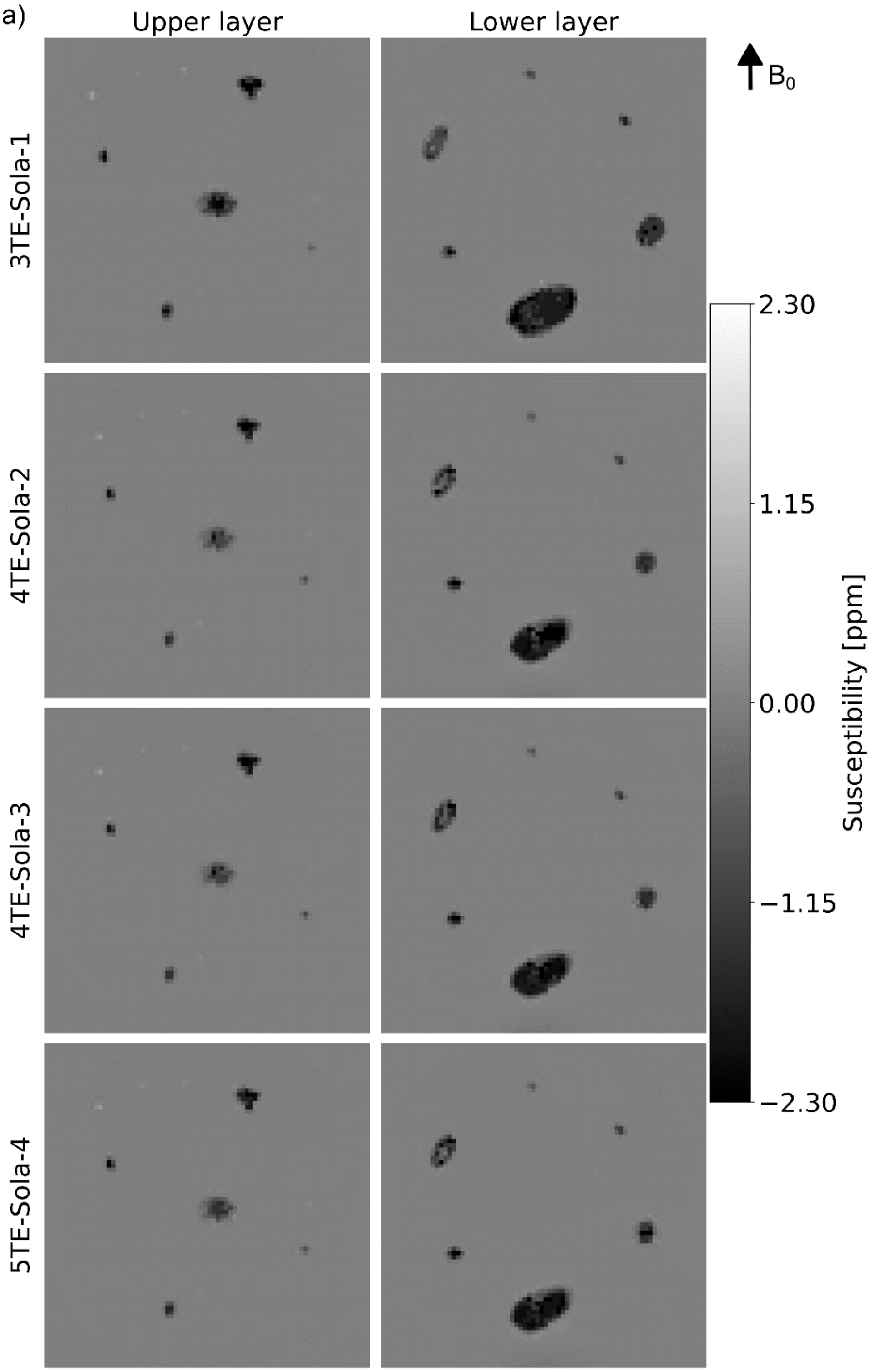

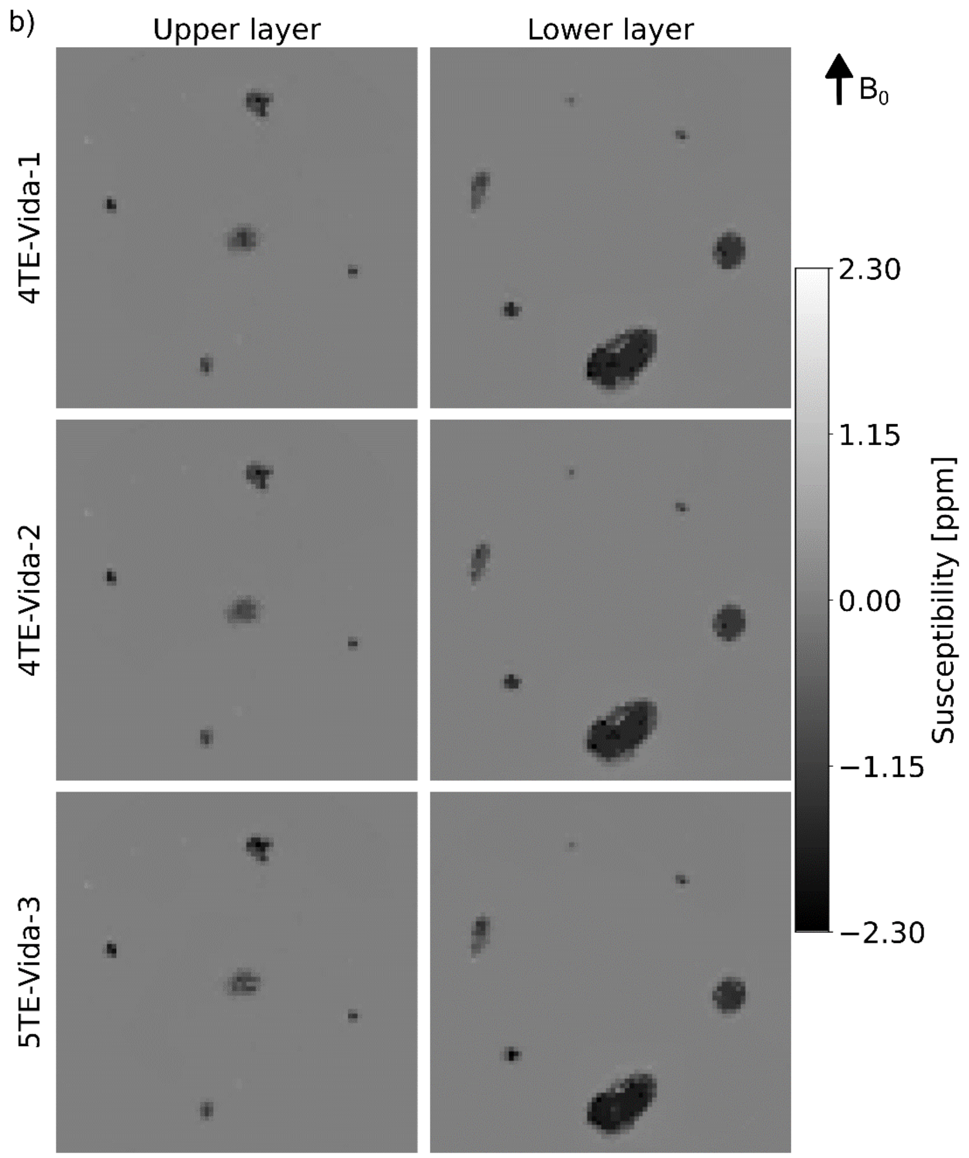
b)
Upper layer
Lower layer
B_0
4TE-Vida-1
4TE-Vida-2
5TE-Vida-3
2.30
1.15
0.00
−1.15
−2.30
Susceptibility [ppm]

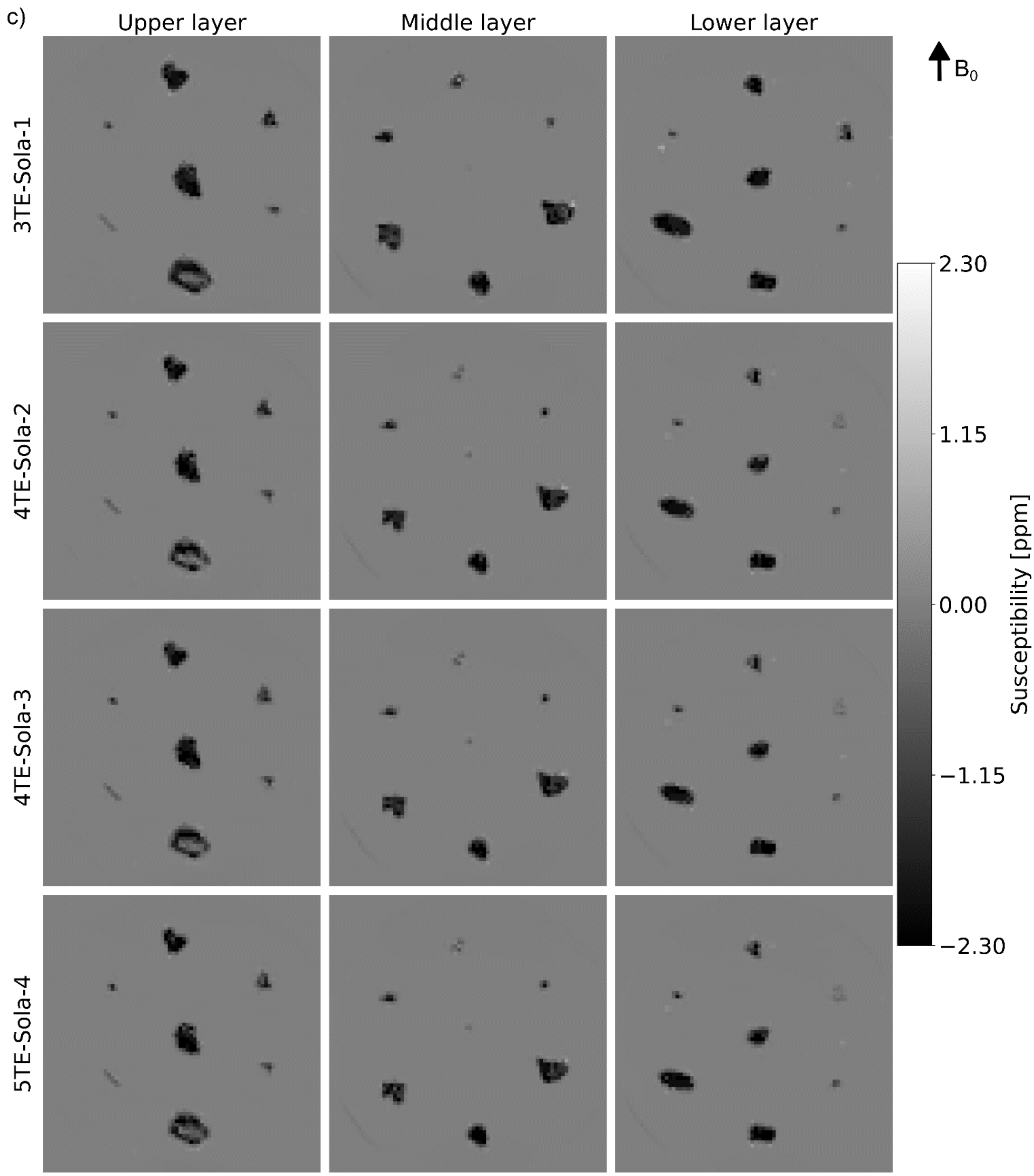

c)
Upper layer
Middle layer
Lower layer
3TE-Sola-1
4TE-Sola-2
4TE-Sola-3
5TE-Sola-4
B_0
2.30
1.15
0.00
−1.15
−2.30
Susceptibility [ppm]

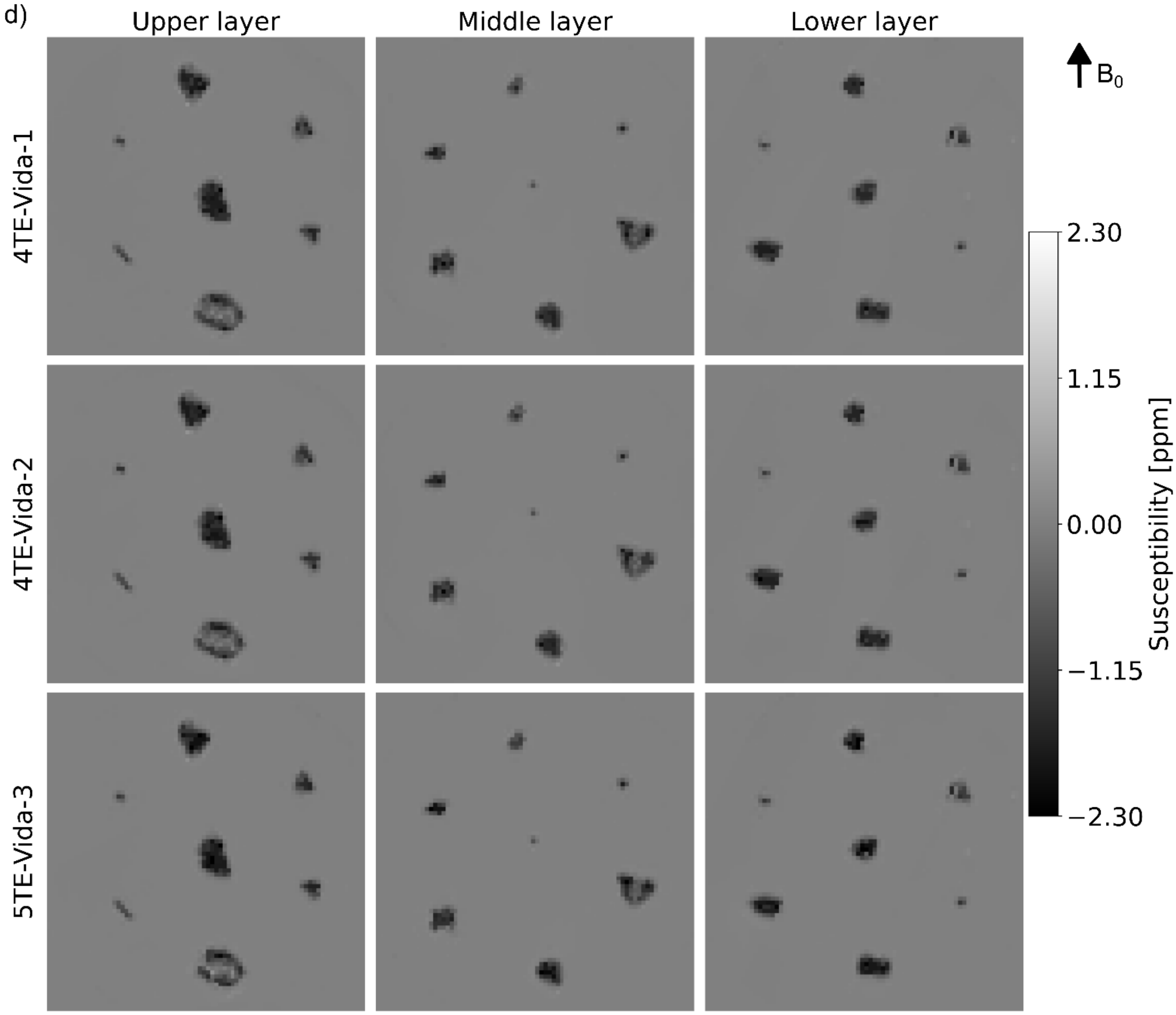

d)
Upper layer
Middle layer
Lower layer
4TE-Vida-1
4TE-Vida-2
5TE-Vida-3
B0
2.30
1.15
0.00
−1.15
−2.30
Susceptibility [ppm]

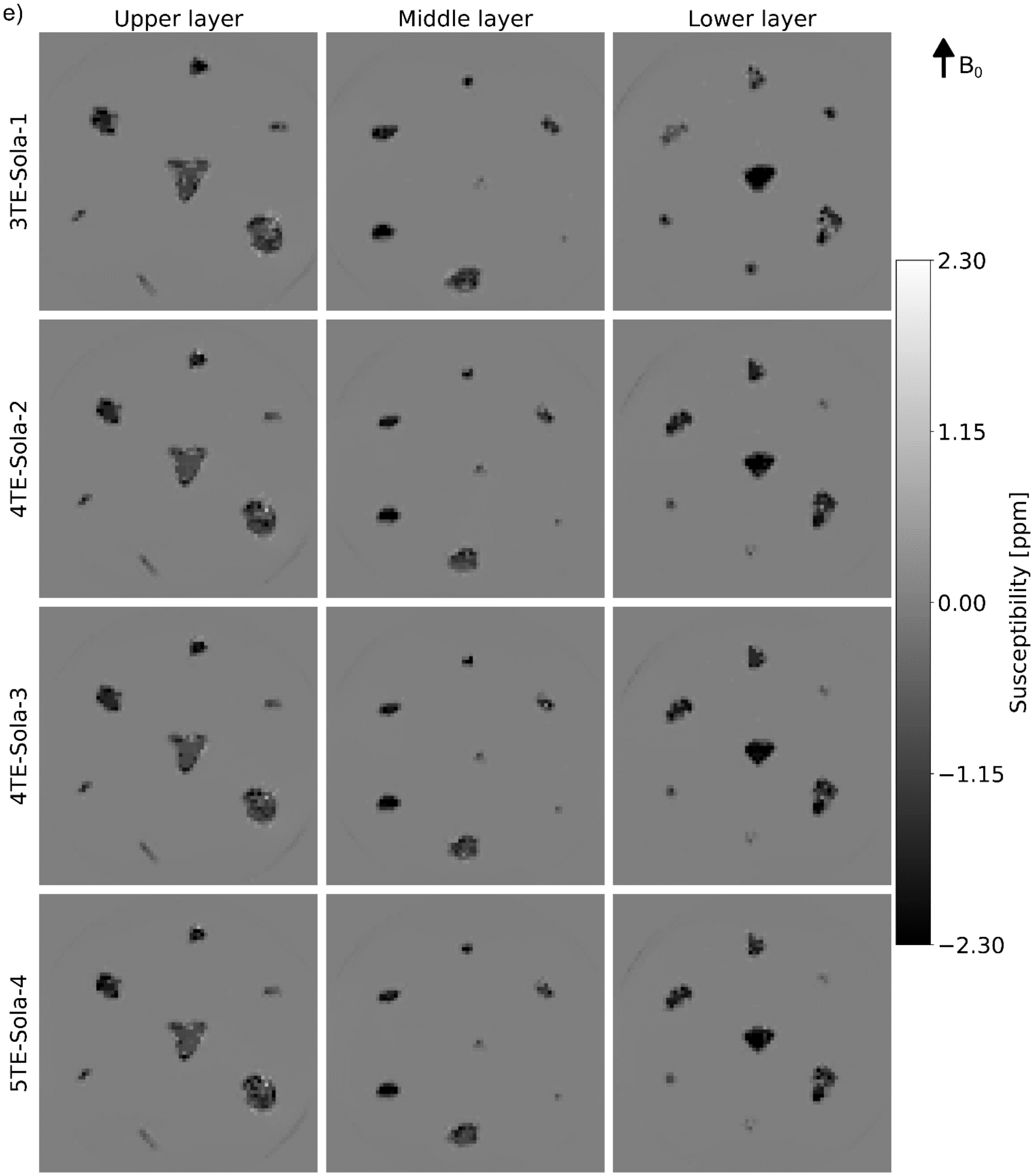

e)
Upper layer
Middle layer
Lower layer
3TE-Sola-1
4TE-Sola-2
4TE-Sola-3
5TE-Sola-4
B0
2.30
1.15
0.00
−1.15
−2.30
Susceptibility [ppm]

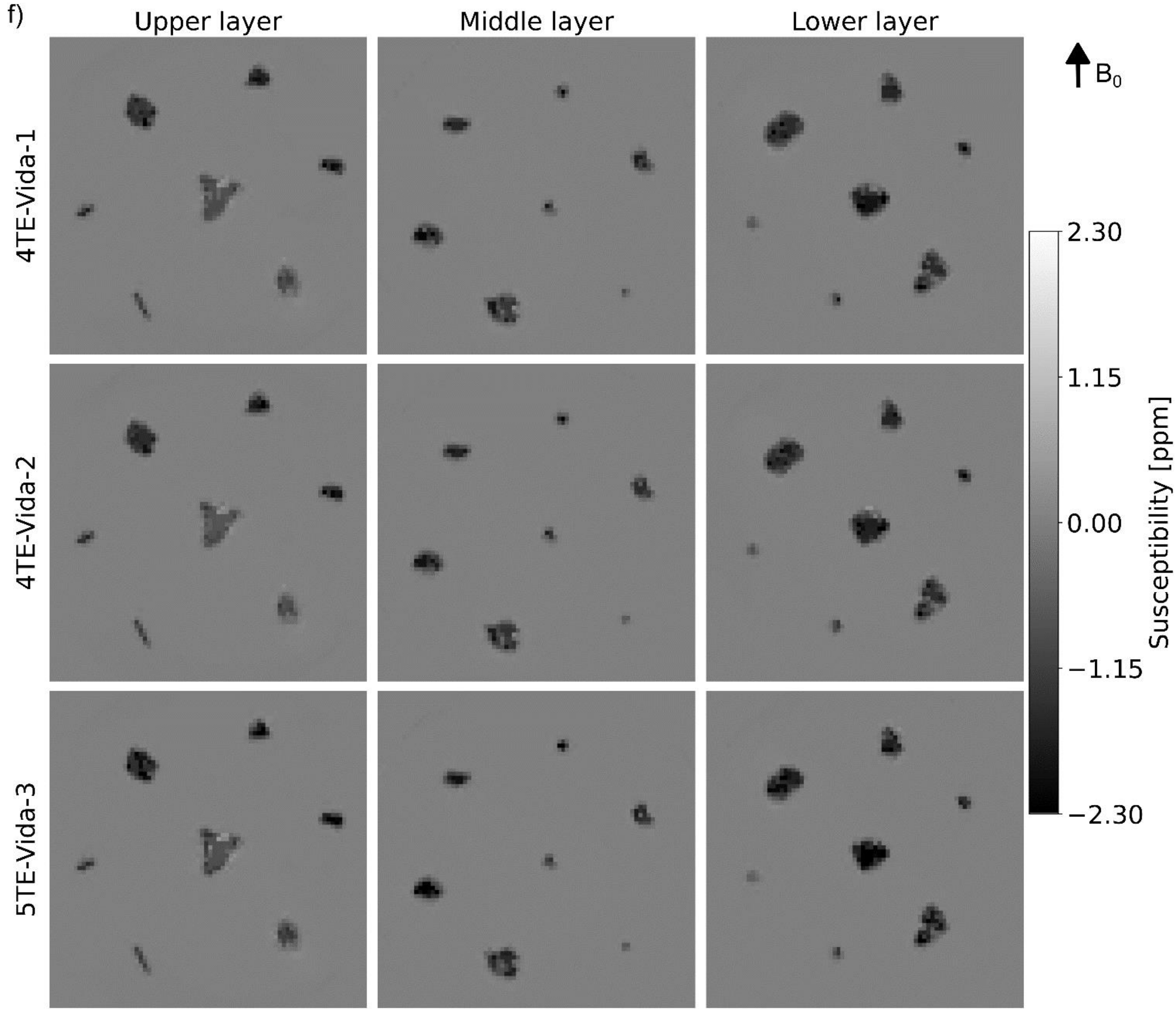

f)
Upper layer
Middle layer
Lower layer
B0
4TE-Vida-1
4TE-Vida-2
5TE-Vida-3
2.30
1.15
0.00
−1.15
−2.30
Susceptibility [ppm]

SUPPORTING INFORMATION FIGURE S2: Mean susceptibility values and SD in [ppm] for the three measured repetitions of all 53 kidney stones, resulting from the acquisition settings **a)** 3TE-Sola-1; **b)** 4TE-Sola-3; **c)** 5TE-Sola-4; **d)** 4TE-Vida-2; and **e)** 5TE-Vida-3. The stones are sorted by diameter in [mm] from smallest to largest along the x-axis, where repetitions of individual kidney stones (KS) are clustered together and labeled with the same ID. Identically colored and shaped markers identify the five major kidney stone types, as well as mixed stones. CaP = carbonate apatite, CaOx = calcium oxalate, UA = uric acid, CY = cystine, ST = struvite, Mixed = mixed stones.

a)

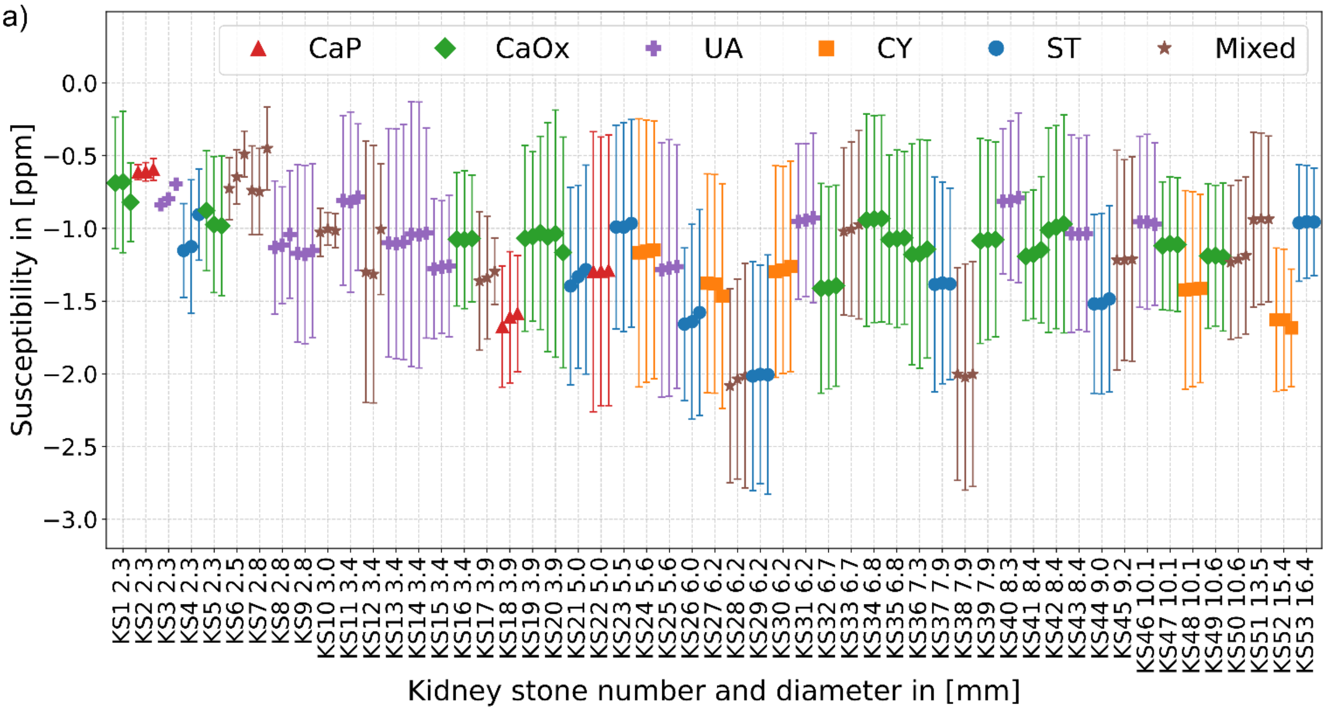

b)

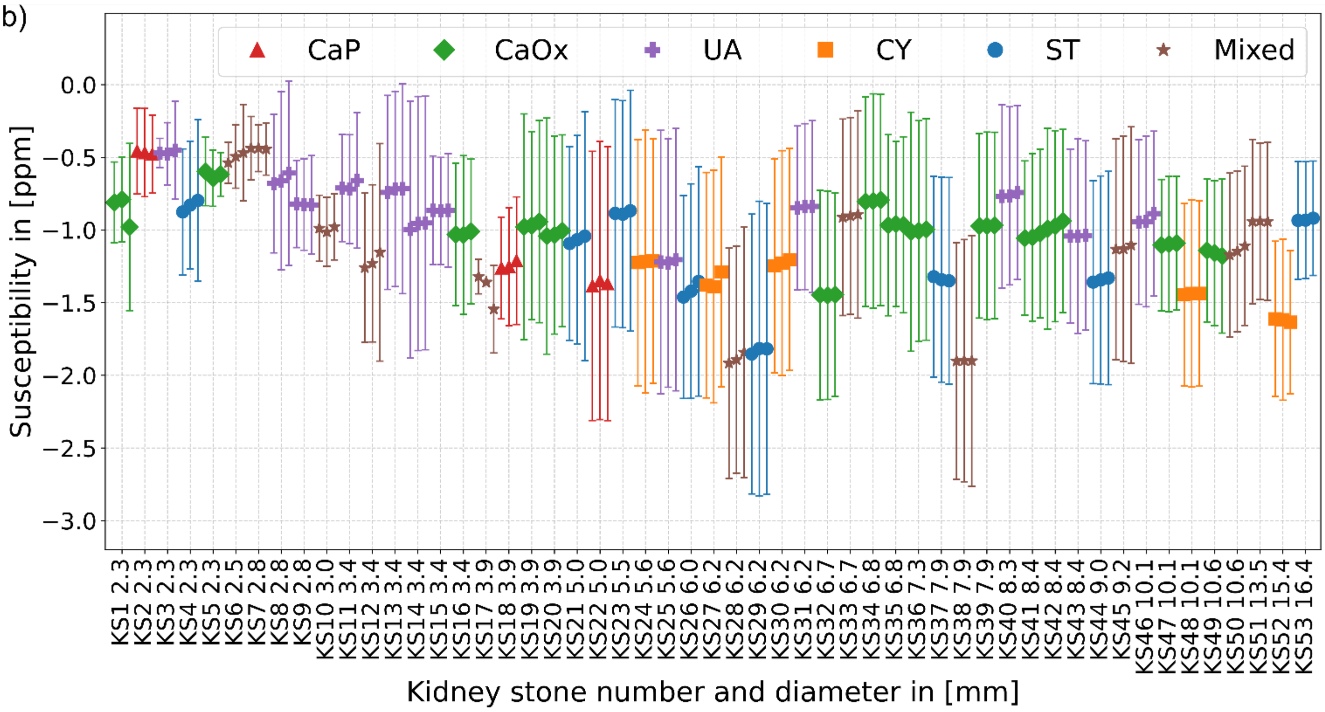

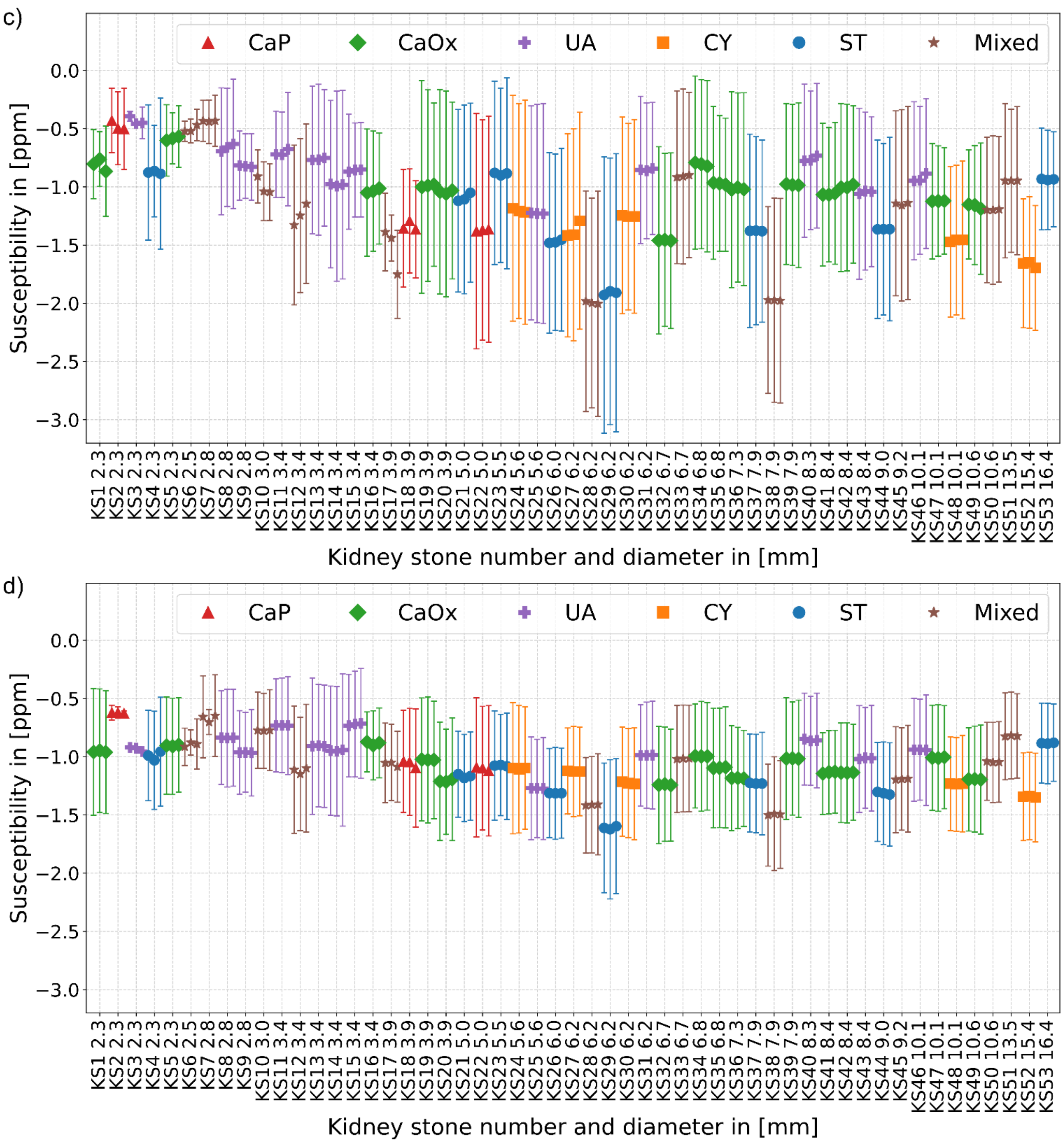
c)
CaP
CaOx
UA
CY
ST
Mixed
Susceptibility in [ppm]
0.0
−0.5
−1.0
−1.5
−2.0
−2.5
−3.0
KS1 2.3, KS2 2.3, KS3 2.3, KS4 2.3, KS5 2.3, KS6 2.5, KS7 2.8, KS8 2.8, KS9 2.8, KS10 3.0, KS11 3.4, KS12 3.4, KS13 3.4, KS14 3.4, KS15 3.4, KS16 3.4, KS17 3.9, KS18 3.9, KS19 3.9, KS20 3.9, KS21 5.0, KS22 5.0, KS23 5.5, KS24 5.6, KS25 5.6, KS26 6.0, KS27 6.2, KS28 6.2, KS29 6.2, KS30 6.2, KS31 6.2, KS32 6.7, KS33 6.7, KS34 6.8, KS35 6.8, KS36 7.3, KS37 7.9, KS38 7.9, KS39 7.9, KS40 8.3, KS41 8.4, KS42 8.4, KS43 8.4, KS44 9.0, KS45 9.2, KS46 10.1, KS47 10.1, KS48 10.1, KS49 10.6, KS50 10.6, KS51 13.5, KS52 15.4, KS53 16.4
Kidney stone number and diameter in [mm]
d)
CaP
CaOx
UA
CY
ST
Mixed
Susceptibility in [ppm]
0.0
−0.5
−1.0
−1.5
−2.0
−2.5
−3.0
KS1 2.3, KS2 2.3, KS3 2.3, KS4 2.3, KS5 2.3, KS6 2.5, KS7 2.8, KS8 2.8, KS9 2.8, KS10 3.0, KS11 3.4, KS12 3.4, KS13 3.4, KS14 3.4, KS15 3.4, KS16 3.4, KS17 3.9, KS18 3.9, KS19 3.9, KS20 3.9, KS21 5.0, KS22 5.0, KS23 5.5, KS24 5.6, KS25 5.6, KS26 6.0, KS27 6.2, KS28 6.2, KS29 6.2, KS30 6.2, KS31 6.2, KS32 6.7, KS33 6.7, KS34 6.8, KS35 6.8, KS36 7.3, KS37 7.9, KS38 7.9, KS39 7.9, KS40 8.3, KS41 8.4, KS42 8.4, KS43 8.4, KS44 9.0, KS45 9.2, KS46 10.1, KS47 10.1, KS48 10.1, KS49 10.6, KS50 10.6, KS51 13.5, KS52 15.4, KS53 16.4
Kidney stone number and diameter in [mm]

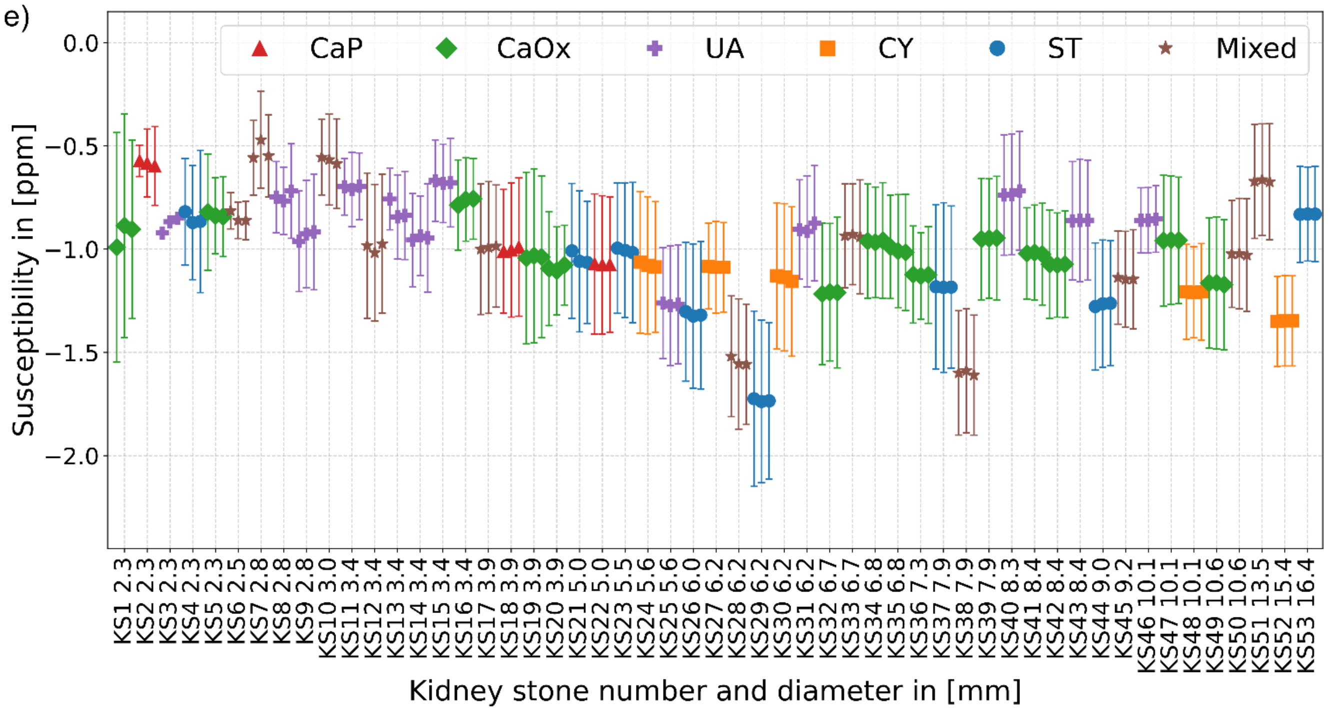
e)
CaP
CaOx
UA
CY
ST
Mixed
Susceptibility in [ppm]
0.0
−0.5
−1.0
−1.5
−2.0
Kidney stone number and diameter in [mm]
KS1 2.3
KS2 2.3
KS3 2.3
KS4 2.3
KS5 2.3
KS6 2.5
KS7 2.8
KS8 2.8
KS9 2.8
KS10 3.0
KS11 3.4
KS12 3.4
KS13 3.4
KS14 3.4
KS15 3.4
KS16 3.4
KS17 3.9
KS18 3.9
KS19 3.9
KS20 3.9
KS21 5.0
KS22 5.0
KS23 5.5
KS24 5.6
KS25 5.6
KS26 6.0
KS27 6.2
KS28 6.2
KS29 6.2
KS30 6.2
KS31 6.2
KS32 6.7
KS33 6.7
KS34 6.8
KS35 6.8
KS36 7.3
KS37 7.9
KS38 7.9
KS39 7.9
KS40 8.3
KS41 8.4
KS42 8.4
KS43 8.4
KS44 9.0
KS45 9.2
KS46 10.1
KS47 10.1
KS48 10.1
KS49 10.6
KS50 10.6
KS51 13.5
KS52 15.4
KS53 16.4

SUPPORTING INFORMATION TABLE S2: Resulting p-value matrices from statistical post-hoc analysis for susceptibility differences between the major kidney stone types CaOx, UA, CY, and ST resulting from statistical analysis of data at **a)** 1.5T, **b)** 3T, and **c)** both field strengths together. The data of each stone type consisted of the mean susceptibility values per individual kidney stone calculated from the three repetitions and respective acquisition settings. Significant differences $(p < 0.05)$ are highlighted in red. CaP was excluded due to its small sample size. CaP = carbonate apatite, CaOx = calcium oxalate, UA = uric acid, CY = cystine, ST = struvite

a)	**CaOx**	**UA**	**CY**	**ST**
**CaOx**	1.0	0.223	0.116	0.432
**UA**	0.223	1.0	**0.003**	0.032
**CY**	0.116	**0.003**	1.0	0.432
**ST**	0.438	0.032	0.432	1.0

b)	**CaOx**	**UA**	**CY**	**ST**
**CaOx**	1.0	0.116	0.193	0.479
**UA**	0.116	1.0	**0.010**	0.070
**CY**	0.193	**0.010**	1.0	0.997
**ST**	0.479	0.070	0.997	1.0

c)	**CaOx**	**UA**	**CY**	**ST**
**CaOx**	1.0	0.124	0.053	0.388
**UA**	0.101	1.0	**0.005**	0.085
**CY**	0.124	**0.005**	1.0	0.992
**ST**	0.388	0.085	0.992	1.0

SUPPORTING INFORMATION FIGURE S3: Mean susceptibility values in ppm of the 53 individual kidney stones in blue for the different acquisition parameter values of the seven acquisition settings on the x-axis for the acquisition parameters **a)** first TE (TE1), **b)** TE spacing (ΔTE), **c)** last TE (TEmax), **d)** flip angle (FA), **e)** field strength. A linear least squares fit applied to the susceptibilities of each acquisition parameter is depicted in orange, with the slope, intercept and coefficient of determination (R2) denoted at the top of each parameter analysis subfigure. Statistically significant differences between susceptibilities of the respective acquisition parameter values are highlighted in black ($*p < 0.01$). Effects of different acquisition parameter values on the kidney stone susceptibilities of the 53 kidney stones were quantified by applying a Friedman test for each acquisition parameter with more than two different parameter values, followed by a Wilcoxon signed-rank test using the Holm-Bonferroni method. For exactly two different acquisition parameter values, a Wilcoxon signed-rank test was calculated. All p-values are listed in Supporting Information Table S3.

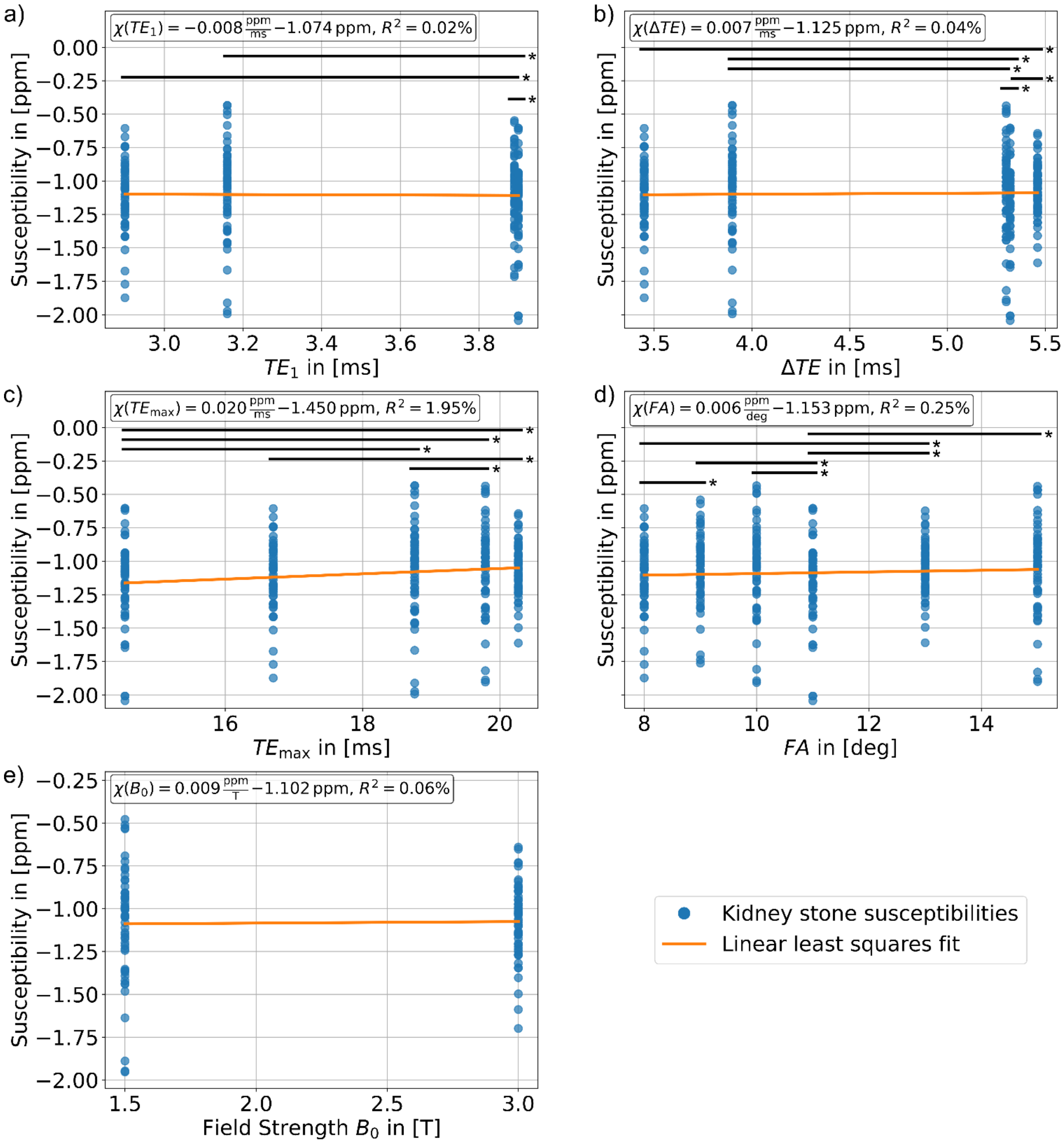

SUPPORTING INFORMATION TABLE S3: Resulting p-value matrices from statistical post-hoc analysis of differences between susceptibility values of the 53 individual kidney stones at the different acquisition parameter values utilized in the seven acquisition settings, together with the median susceptibility values (Median χ) for each acquisition parameter setting. The p-value matrices are given for the parameters **a)** first TE (TE_1), **b)** TE spacing (ΔTE), **c)** last TE (TE_{max}), **d)** flip angle (FA), **e)** field strength. Significant differences ($p < 0.05$) are highlighted in red.

a) **TE_1**	**2.90 ms**	**3.16 ms**	**3.89 ms**	**3.90 ms**
**2.90 ms**	1.0	0.174	**2.08×10^{-5}**	0.189
**3.16 ms**	0.174	1.0	0.362	**2.43×10^{-5}**
**3.89 ms**	**2.08×10^{-5}**	0.362	1.0	**4.20×10^{-7}**
**3.90 ms**	0.189	**2.43×10^{-5}**	**4.20×10^{-7}**	1.0
**Median χ**	-1.07 ppm	-1.02 ppm	-1.03 ppm	-1.10 ppm

b) **ΔTE**	**3.45 ms** -1.07 ppm	**3.90 ms**	**5.30 ms**	**5.32 ms**	**5.46 ms**
**3.45 ms**	1.0	0.233	0.053	0.283	**1.62×10^{-7}**
**3.90 ms**	0.233	1.0	**5.93×10^{-5}**	**4.86×10^{-5}**	1.0
**5.30 ms**	0.053	**5.93×10^{-5}**	1.0	**2.67×10^{-6}**	1.0
**5.32 ms**	0.283	**4.86×10^{-5}**	**2.67×10^{-6}**	1.0	**6.57×10^{-4}**
**5.46 ms**	**1.62×10^{-7}**	1.0	1.0	**6.57×10^{-4}**	1.0
**Median χ**	-1.07 ppm	-1.02 ppm	-1.01 ppm	-1.10 ppm	-1.04 ppm

c) **TE_{max}**	**14.54 ms**	**16.70 ms**	**18.76 ms**	**19.79 ms**	**20.27 ms**
**14.54 ms**	1.0	0.283	**4.86×10^{-5}**	**2.67×10^{-6}**	**6.57×10^{-4}**
**16.70 ms**	0.283	1.0	0.233	0.053	**1.62×10^{-7}**
**18.76 ms**	**4.86×10^{-5}**	0.233	1.0	**5.93×10^{-5}**	1.0
**19.79 ms**	**2.67×10^{-6}**	0.053	**5.93×10^{-5}**	1.0	1.0
**20.27 ms**	**6.57×10^{-4}**	**1.62×10^{-7}**	1.0	1.0	1.0
**Median χ**	-1.10 ppm	-1.07 ppm	-1.02 ppm	-1.01 ppm	-1.04 ppm

d) **FA**	**8°**<br>- 1.07 ppm	**9°**	**10°**	**11°**	**13°**	**15°**
**8°**	1.0	**4.07×10^{-4}**	0.095	0.566	**8.61×10^{-7}**	0.069
**9°**	**4.07×10^{-4}**	1.0	1.0	**1.45×10^{-6}**	1.0	1.0
**10°**	0.095	1.0	1.0	**5.94×10^{-6}**	1.0	0.095
**11°**	0.566	**1.45×10^{-6}**	**5.94×10^{-6}**	1.0	**0.001**	**2.90×10^{-6}**
**13°**	**8.61×10^{-7}**	1.0	1.0	**0.001**	1.0	1.0
**15°**	0.069	1.0	0.095	**2.90×10^{-6}**	1.0	1.0
**Median χ**	-1.07 ppm	-1.03 ppm	-1.02 ppm	-1.10 ppm	-1.04 ppm	-1.00 ppm

e) **Field strength**	**1.5 T**	**3 T**
**1.5 T**	1.0	0.791
**3 T**	0.791	1.0
**Median χ**	-1.04 ppm	-1.06 ppm

SUPPORTING INFORMATION SIMULATIONS

Purpose

To investigate and quantify different QSM reconstruction approaches in kidney stone regions characterized by high amounts of propagated phase noise. This is achieved by using forward simulations of a numerical kidney stone phantom with a known susceptibility ground truth.

Methods

Susceptibility ground truth

A numerical 3D kidney stone susceptibility phantom was generated in python (version 3.11), based on the segmentations and susceptibility data of the first repetition of Phantom 2 for the acquisition setting 4TE-Sola-1.

To this end, the volume of interest (VOI) of the binary mask of Phantom 2 was assigned a susceptibility value of $\chi_{\mathrm{Agar-Agar}} = 0.004\ \mathrm{ppm}$, which was found during preliminary testing. All 21 kidney stone regions – belonging to the stones embedded in Phantom 2 – were then assigned individual susceptibility values using the stone segmentations created for phantom data evaluation. These individual kidney stone susceptibility values were in the range of $\chi_{\mathrm{stones}} \in [-0.67, -1.30]\ \mathrm{ppm}$ and were obtained by preliminary QSM reconstruction tests of the acquired phantom data. Finally, the susceptibility values outside of the VOI of Phantom 2 were set to $\chi_{\mathrm{background}} = 0\ \mathrm{ppm}$. The resulting susceptibility map of the numerical kidney stone phantom constituted the susceptibility ground truth (see Figure R1, bottom row).

Local field map calculation

A 3D local field map $\Delta f(\boldsymbol{r})$ of the numerical phantom was calculated from the ground truth susceptibility map $\chi(\boldsymbol{r})$ using the convolution

$$\Delta f(\boldsymbol{r}) = \frac{\gamma}{2\pi} \cdot B_0 \cdot \left(\chi(\boldsymbol{r}) * d_z(\boldsymbol{r})\right) \quad ,$$

with the dipole kernel $d_z(\boldsymbol{r})$, the gyromagnetic ratio $\gamma = 2.67 \times 10^8\ \frac{\mathrm{Hz}}{\mathrm{T}}$, and the main magnetic field strength $B_0 = 3\ \mathrm{T}$.[1,2] The resulting local field map served as one of three variants, where it represented an unmodified local field map with perfect frequency information inside stone regions (Variant 1). The second local field map variant was generated by setting the kidney stone regions in the local field map to zero before dipole inversion (Variant 2). This QSM reconstruction approach aimed to reduce the high noise levels observed inside most kidney stones in the local field map, since this noise did not reflect the susceptibility of the kidney stones, but rather resulted from propagated phase noise due to a lack of protons inside the stones.[3,4] For the third variant, the values of the local field map inside the stone regions were replaced by Gaussian noise (Variant 3). This represented a conventional QSM reconstruction approach, where the dipole inversion is applied directly on local field maps of measured kidney stone data containing propagated phase noise due to low signal within the stones.[3-5] The local field values containing Gaussian noise were modelled based on the measured distribution of local field values within kidney stone regions using a mean value of $\mu_{\mathrm{noise}} = -10.86\ \mathrm{Hz}$ and standard deviation of $\sigma_{\mathrm{noise}} = 39.36\ \mathrm{Hz}$. The three resulting local field map variants are displayed in the right column of Figure R1.

Dipole inversion

For all three local field map variants, the corresponding susceptibility maps were calculated in MATLAB (R2022b; MathWorks, Natick, USA) utilizing the SEPIA toolbox[6] (version 1.2.1.1).[2] Dipole inversion was performed using the algorithm MEDI+0[7] with a lambda factor of 50, and lambda CSF – a regularization parameter enforcing homogeneous susceptibility distribution in CSF regions – deactivated. Additional inputs for the MEDI+0 algorithm consisted of the

previously generated binary mask of Phantom 2, and the previously acquired magnitude image of the ex-vivo Phantom 2 of the acquisition setting 4TE-Sola-1.

Evaluation
For qualitative comparison, the susceptibility maps were visually inspected. For quantitative evaluation, mean susceptibility values and standard deviations were calculated for each of the 21 individual kidney stone per simulated reconstruction approach using the previously generated manual kidney stone segmentations.

Results
Figure R1 depicts the three generated local field maps and corresponding susceptibility maps resulting from the dipole inversion, together with the simulated ground truth of the numerical kidney stone phantom. The susceptibility map of Variant 2, which was reconstructed from the local field map with kidney stone regions set to zero, showed a substantial reduction in susceptibility inhomogeneities inside the stone regions compared to susceptibility maps resulting from local field maps with Gaussian noise within kidney stone regions. Additionally, paramagnetic regions located at the boundary between kidney stones and Agar-Agar medium in the susceptibility map were also less pronounced in Variant 2 compared to the data underlying Gaussian noise of Variant 3. Nonetheless, both the paramagnetic regions at the stone edge and susceptibility inhomogeneities within stones were still visible in the susceptibility map of Variant 2. In addition, the individual kidney stone susceptibilities of the reconstruction approach Variant 2 with stone regions set to zero in the local field map seemed to slightly deviate from both the simulated ground truth susceptibilities and kidney stone susceptibilities of reconstructed from the unmodified local field maps of Variant 1. In contrast, susceptibility maps reconstructed with Variant 1 showed only negligible susceptibility inhomogeneities within kidney stone regions, as well as no noticeable deviation in mean susceptibility for the kidney stones. Susceptibility maps reconstructed with Variant 3 displayed the highest amount of noisy inhomogeneities within kidney stone regions.
Figure R2 displays the quantitative analysis of the mean susceptibility values belonging to the 21 individual kidney stones. For the three investigated reconstruction approaches (differently colored), the respective standard deviation of susceptibilities within each kidney stone is depicted. The mean kidney stone susceptibilities reconstructed from the unmodified local field map of Variant 1 and the local field map with additional Gaussian noise of Variant 3 showed similarly close agreement with the simulation ground truth. In contrast, the mean susceptibility values of kidney stones reconstructed with Variant 2 resulted in susceptibilities considerably closer to 0 ppm compared to the ground truth, especially for larger kidney stones. Variant 3 yielded substantially larger standard deviations, which is consistent with the observations in Figure R1. For Variant 2, the standard deviations of susceptibility within kidney stone regions were just slightly larger than those resulting from the unmodified local field map of Variant 1.

Conclusion
The conventional QSM reconstruction approach Variant 3, where the dipole inversion was applied directly on local field maps containing propagated phase noise in kidney stone regions, resulted in mean susceptibility values close to the simulated ground truth. In contrast, Variant 2 of setting kidney stone regions in the local field map to zero before dipole inversion was shown to result in an unreliable underestimation of mean susceptibility values of the simulated kidney stones. Although the approach of Variant 2 substantially reduced susceptibility inhomogeneity inside the kidney stones, using a conventional QSM reconstruction approach even on local field maps with Gaussian noise in stone regions proved more reliable for quantitative susceptibility estimation of kidney stones.

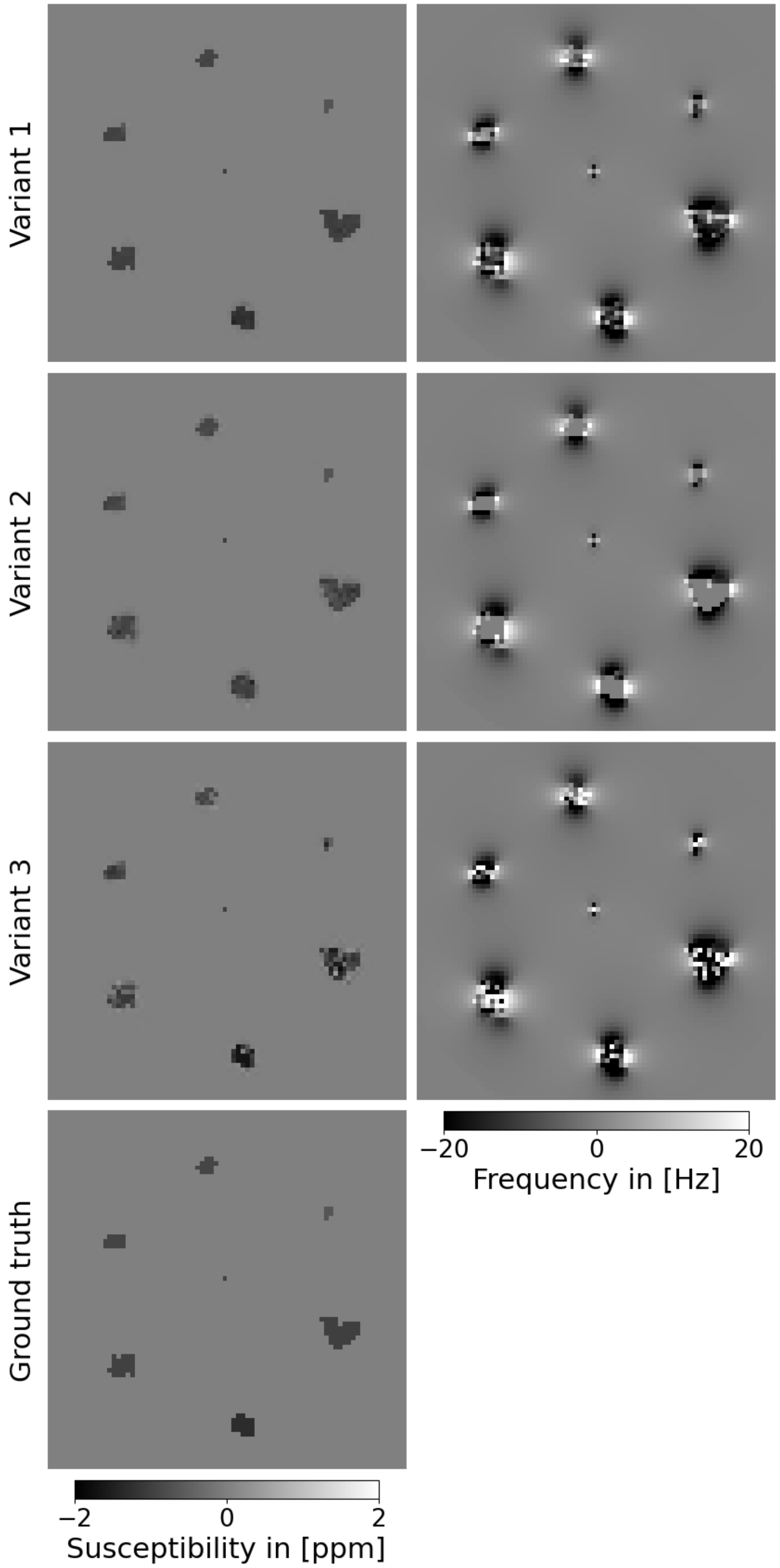

Figure R1: Representative simulated local field maps (right column) and susceptibility maps (left column) of the numerical kidney stone phantom depicting seven kidney stones. Shown are the generated simulation ground truth (bottom), the two simulated reconstruction approaches Variant 2 and Variant 3 for handling propagated phase noise in kidney stone regions (middle), and the simulated – but in reality unfeasible – QSM reconstruction Variant 1 with perfect frequency information inside stone regions (top).

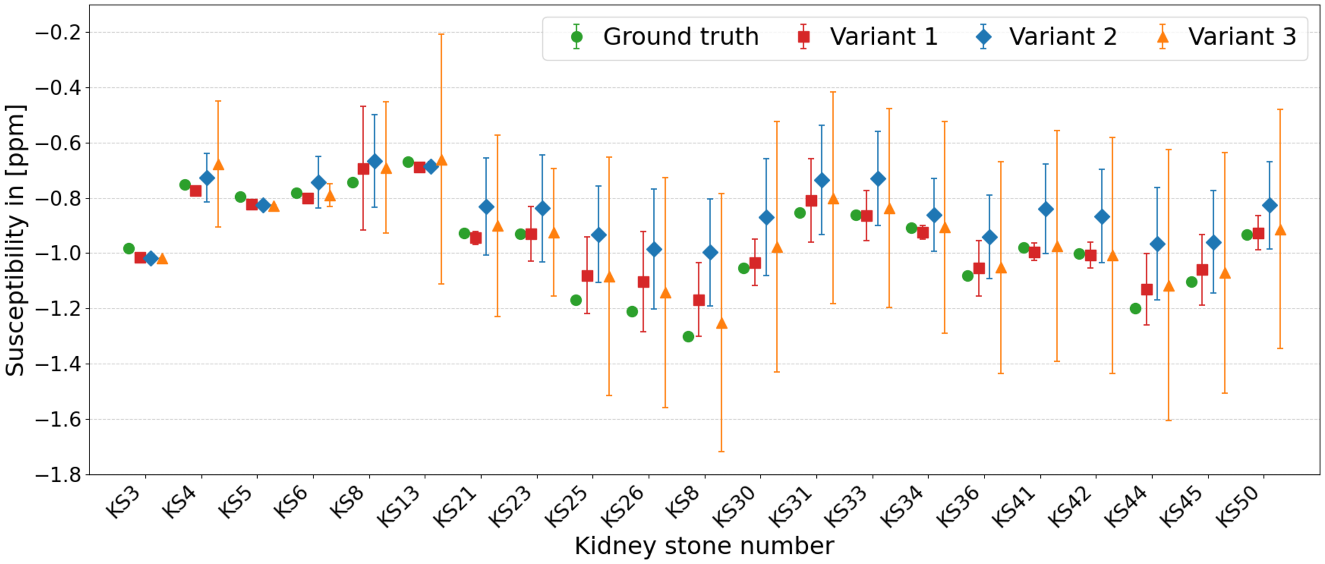

Figure R2: Mean susceptibility values and SD in [ppm] of the 21 synthetic kidney stone regions inside the numerical phantom. Differently colored and shaped markers represent the four different susceptibility maps. Along the x-axis are the individual kidney stones (KS), with labels corresponding to the respective stones in the main manuscript.

References

1 Deistung, A., Schweser, F. & Reichenbach, J. R. Overview of quantitative susceptibility mapping. *NMR Biomed* **30** (2017). https://doi.org/10.1002/nbm.3569

2 Committee, Q. S. M. C. O. *et al.* Recommended implementation of quantitative susceptibility mapping for clinical research in the brain: A consensus of the ISMRM electro-magnetic tissue properties study group. *Magn Reson Med* **91**, 1834–1862 (2024). https://doi.org/10.1002/mrm.30006

3 Cheng, P. M., Moin, P., Dunn, M. D., Boswell, W. D. & Duddalwar, V. A. What the radiologist needs to know about urolithiasis: part 1--pathogenesis, types, assessment, and variant anatomy. *AJR Am J Roentgenol* **198**, W540–547 (2012). https://doi.org/10.2214/AJR.10.7285

4 Kalb, B. *et al.* Acute abdominal pain: is there a potential role for MRI in the setting of the emergency department in a patient with renal calculi? *J Magn Reson Imaging* **32**, 1012–1023 (2010). https://doi.org/10.1002/jmri.22337

5 Aja-Fernández, S. & Vegas-Sánchez-Ferrero, G. Statistical analysis of noise in MRI. *Switzerland: Springer International Publishing* (2016).

6 Chan, K. S. & Marques, J. P. SEPIA-Susceptibility mapping pipeline tool for phase images. *Neuroimage* **227**, 117611 (2021). https://doi.org/10.1016/j.neuroimage.2020.117611

7 Liu, Z., Spincemaille, P., Yao, Y., Zhang, Y. & Wang, Y. MEDI+0: Morphology enabled dipole inversion with automatic uniform cerebrospinal fluid zero reference for quantitative susceptibility mapping. *Magn Reson Med* **79**, 2795–2803 (2018). https://doi.org/10.1002/mrm.26946
